# Cobalt Oxide (Co_3_O_4_) Thin Films Synthesized by Atmospheric Pressure PECVD: Deposition Mechanisms and Catalytic Potential

**DOI:** 10.1021/acsaem.5c00280

**Published:** 2025-05-29

**Authors:** João Mallmann, Jean-Baptiste Chemin, Drialys Cardenas Morcoso, Adrian-Marie Philippe, Simon Bulou, Nihed Chaabane, Fabien Rouillard, Patrick Choquet, Nicolas D. Boscher

**Affiliations:** † 87145 Luxembourg Institute of Science and Technology, Advanced Plasma and Vapor Deposition Processes Engineering, L-4362 Esch-sur-Alzette, Luxembourg; # Luxembourg Institute of Science and Technology, Advanced Characterization of Surface, Interface and Structure, L-4422 Belvaux, Luxembourg; ‡ CEA, Service de Recherche en Corrosion et Comportement des Matériaux, Université Paris Saclay, 91191 Gif-sur-Yvette, France; § CEA, Institut National des Sciences et Techniques Nucléaires, Université Paris Saclay, 91191 Gif-sur-Yvette, France

**Keywords:** atmospheric plasma deposition, Co_3_O_4_ thin film, chemical vapor deposition, growth mechanisms, electrocatalysis, oxygen evolution reaction

## Abstract

Crystalline and electrocatalytically active cobalt oxide (Co_3_O_4_) thin films were successfully synthesized under open-air conditions using atmospheric pressure plasma-enhanced chemical vapor deposition (AP-PECVD) with the Co­(acac)_3_ precursor. This study explored the influence of process parameters on the composition, crystallinity, and quality of the resulting thin films. It was found that the substrate temperature had a negligible effect due to the inherent heating by the plasma afterglow. The presence of atmospheric oxygen was identified as crucial for forming Co_3_O_4_ thin films and eliminating residual impurities such as carbon and nitrogen, as demonstrated by experiments in O_2_-free environments. The formation of Co_3_O_4_ was attributed to radical-mediated reactions, where the reactive species generated in the plasma interacted with oxygen-rich molecules from the surrounding air. These findings provide valuable insights into the deposition mechanisms and catalytic potential of Co_3_O_4_ thin films synthesized via AP-PECVD.

## Graphical abstract

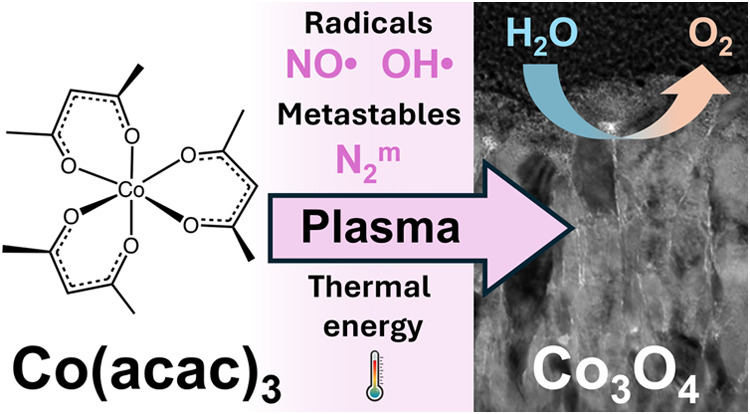

## 1 Introduction

Catalytic processes, including electrocatalysis, offer environmentally sustainable solutions for minimizing harmful pollutants and reducing greenhouse gas emissions. Its efficiency is especially notable in renewable energy technologies, including hydrogen generation via water splitting, simple and low-energy molecule conversion into complex chemicals, and biomass transformation into biofuels.^1^ Finding alternatives to costly and rare materials, including noble metals such as RuO_2_ and IrO_2_, is crucial. Co_3_O_4_ provides a more affordable alternative with an efficient oxygen evolution reaction (OER) activity^1–7^ and promising catalytic potential for energy storage and chemical synthesis.^8^

Co_3_O_4_ has a spinel structure known as AB_2_O_4_, where Co^2+^ occupies tetrahedral sites (A) and Co^3+^ occupies octahedral sites (B).^9,10^ According to Cho et al., Co_3_O_4_ demonstrates self-doping effects that significantly influence its OER activity. This behavior is associated with the Jahn–Teller effect, which plays a crucial role in modifying the electronic structure and catalytic performance of such materials.^4^ However, the exact activity of each site remains debatable. While some consider Co^2+^ ions as the main catalytic active sites due to the increase in oxygen vacancies,^11^ others identified superior activity for Co^3+^ octahedral sites, considering they increase adsorption, desorption, and activation of oxygen species.^12^ Besides, the Co_3_O_4_ catalyst undergoes further oxidation under an OER alkaline environment.^7^ Indeed, Co_3_O_4_ oxidizes on its outer surface during the electrocatalytic process to form CoOOH, which subsequently oxidizes to CoO_2_ upon further increase of the applied potential, suggesting that Co^4+^ is essential for increased electrocatalytic activity. Yet, Co_3_O_4_ remains the most stable CoO_*x*_ phase with a high oxidation state.^5,7^

For practical applications, including heterogeneous electrocatalysis, it is desirable to use Co_3_O_4_ in thin film form. These thin films have been prepared by various methods such as chemical vapor deposition (CVD),^13^ atomic layer deposition (ALD),^14^ sol–gel,^15^ electrodeposition,^5^ physical vapor deposition (PVD),^16^ plasma spraying,^17^ plasma-enhanced CVD (PECVD),^18^ and MOCVD.^19^ Among the developed routes, low-pressure PECVD involved the spraying of an aqueous solution of cobalt nitrate into a radio frequency (RF) low-pressure plasma discharge (40 MHz, 6 mbar) to form Co_*x*_O_*y*_ thin films that were converted into crystalline Co_3_O_4_ after annealing.^18^ On the other hand, an attempt to spray a solution of cobalt carbonyl dissolved in hexene into a capacitive coupled external electrode RF plasma reactor (13.56 MHz, 1 mbar) yielded amorphous cobalt oxide in a plasma polymer thin film.^18^

Atmospheric pressure PECVD (AP-PECVD) is a promising technique for producing crystalline oxides, such as TiO_2_,^20,21^ ZnO,^22^ and SrTiO_3_ ^23^ directly in thin film form on various substrates.^24^ The AP-PECVD processes stand out for their ability to work at atmospheric pressure on large and geometrically complex substrates, and ease of implementation and scaling-up for industrial applications.^22,25,26^ AP-PECVD processes generate reactive species formed from the ionization of a plasma gas whose composition is determined by the targeted reaction, e.g., polymerization,^27^ reduction^28^ or oxidation.^21^ Among the reactive species composing atmospheric plasmas, a range of highly energetic species, with energies greater than 10 eV, can be used to activate and clean surfaces, as well as trigger chemical reactions with metal salts, organic^27^ and organometallic precursors^23^ to produce functional coatings. These reactions include the fragmentation of the thin film precursor upon exposure to plasma discharge or afterglow and chemical reactions involving the plasma reactive species and the thin film precursor.^25,29,30^ Interestingly, AP-PECVD processes allow the use of a wide spectrum of thin film precursors since they can be injected in the form of vapors,^27^ aerosols,^23^ or directly deposited on a surface as liquid layers before plasma curing.^28^

Nonetheless, atmospheric plasma deposition presents several challenges compared to other low-pressure techniques, particularly concerning the synthesis of dense and uniform films with minimal residual impurities and without particle formation. Numerous studies have focused on understanding the parameters and mechanisms that promote thin film growth by AP-PECVD in order to expand its industrial applicability.^22,25,26,29^ Merche et al.^25^ emphasized the significance of precursor injection in the discharge and afterglow regions. When injected directly into the discharge region, precursors are exposed to electrons, ions, radicals, and radiation, resulting in their rapid decomposition, which increases the likelihood of gas-phase reactions leading to undesirable powder formation.^26^ However, such powder formation can be mitigated by controlling the input plasma power, dilution gas, and precursor concentration, with respect to the precursor’s reactivity, or by injecting precursors into the afterglow region. Massines et al.^29^ examined the role of plasma in the thin film deposition processes, noting that at atmospheric pressure, the low mean free path causes kinetic energy losses of electrons and ions due to increased collisions with heavier gas particles, thereby raising the plasma gas temperature and decreasing the concentration of these species. Consequently, the primary energetic contribution arises from metastable states (e.g., N_2_*), which can interact with neutral precursor molecules, and plays a significant role in precursor activation and thin film deposition.^31^ However, the quenching of metastable species, when encountering, for instance, atmospheric O_2_ or the chemical precursor, rapidly decreases their concentrations by creating not only relatively stable species but also atomic and molecular radicals.^32^ The subsequent recombination of these radicals with other reactive species, ions, or other radicals is critical for thin film nucleation and growth.^22,33^ Finally, nucleation and growth must primarily occur at the gas/substrate interface to ensure dense film production.^25^ Some thin film growth pathways were discussed. However, plasma chemistry is highly complex, and numerous factors influence the growth mechanisms of thin films by AP-PECVD.

The present work aims to synthesize an electrocatalytically active cobalt oxide (Co_3_O_4_) thin film using a scalable AP-PECVD approach and to investigate the deposition mechanism through variation of the AP-PECVD parameters. The formation of a dense and homogeneous Co_3_O_4_ layer characterized by low impurity content and reduced powder formation was demonstrated using transmission electron microscopy (TEM), X-ray photoelectron spectroscopy (XPS), Raman spectroscopy, X-ray diffraction (XRD), and secondary ion mass spectrometry (SIMS). The influence of the carrier gas, substrate heating temperature, and surrounding atmosphere on the thin film composition and structure is assessed. Finally, the potential of the Co_3_O_4_ thin film for electrocatalytic applications is demonstrated using cyclic voltammetry and chronoamperometry for the electrocatalytic oxygen evolution reaction (OER).

## 2 Experimental Section

### 2.1 Atmospheric Pressure Plasma-Enhanced Chemical Vapor Deposition

Figure 1 illustrates a schematic of the AP-PECVD system based on a blown arc discharge device, also called a plasma torch (ULS Omega), commercialized by AcXys Technologies. The generation of N_2_ plasma is based on the ignition of an electric arc between two concentric electrodes, a central high-voltage electrode and an outer ground electrode, by means of a 100 kHz sinusoidal high voltage (1000 W). To stabilize this arc discharge, a vortex is created along the two electrodes by a 50 L·min^–1^ N_2_ gas flow, which causes the arc to rotate around the central high-voltage electrode and finally be blown outside the nozzle.

**Figure 1:**
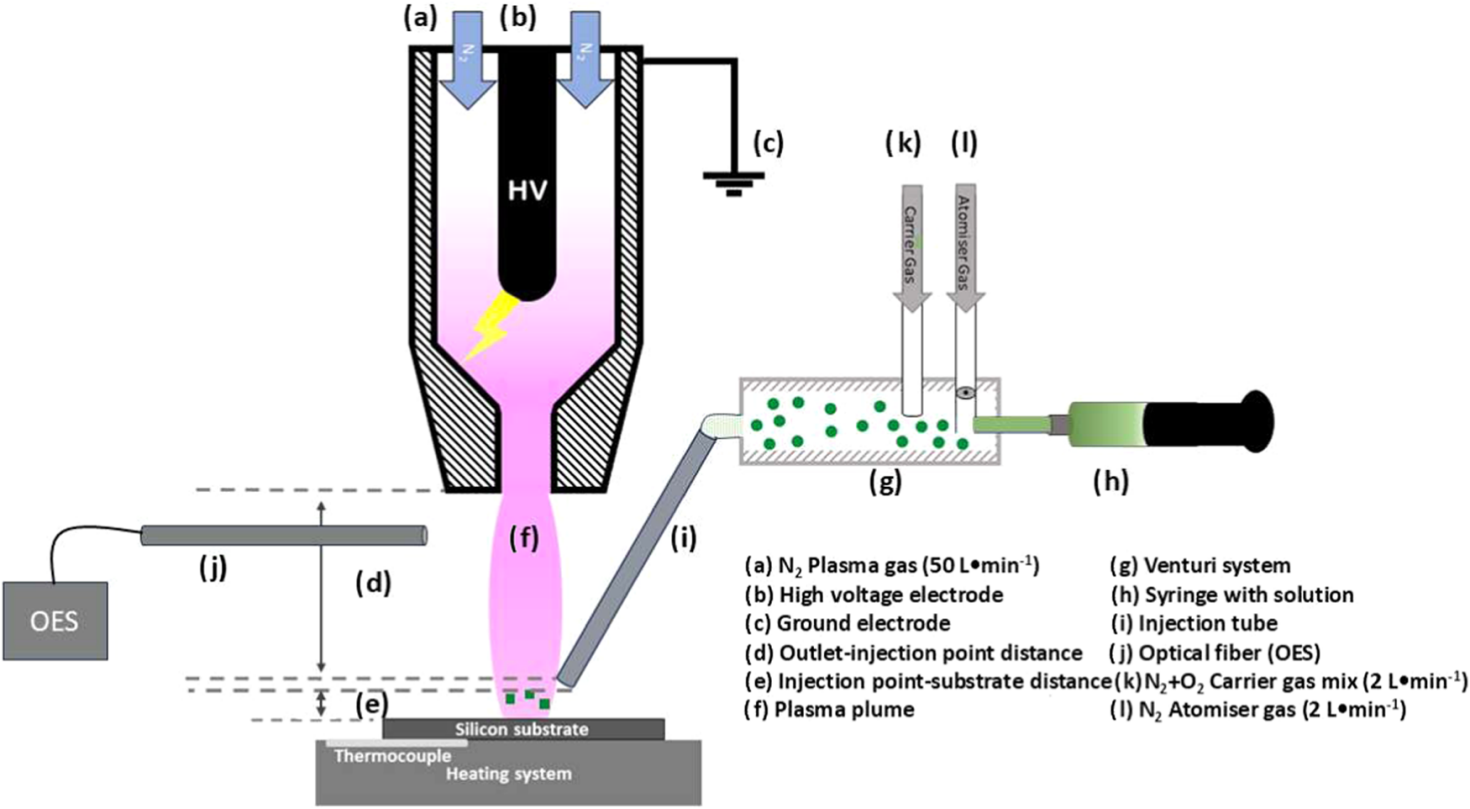
Schematic representation of the experimental setup used for the preparation of Co_3_O_4_ thin films. The blown arc discharge device is mounted on a 6-axis robotic arm, which allows operation in the dynamic mode.

The precursor solution, which comprises a cobalt precursor cobalt acetylacetonate (Co­(acac)_3_) and a solvent (acetone, ethanol, isopropanol, or water), is injected at a rate of 400 μL·min^–1^ into the plasma afterglow in the form of an aerosol generated by an atomizing system based on the Venturi effect. This system generates a high-speed gas flow (2 L·min^–1^) that breaks the solution into micrometer-sized droplets (ranging from 1–5 μm). The aerosol is then conducted in the plasma afterglow region through a 1/8 in. stainless steel tube using a total gas flow of 4 L·min^–1^ comprising the carrier gas (N_2_ and/or O_2_) and atomizing gas (N_2_). The tube’s outlet remains 1 mm above the substrate, while the plasma nozzle is positioned 15 mm above the injection tube. The total distance from the plasma nozzle to the substrate is thus 17.5 nm since it considers (d) + (e) + the injection tube.

The plasma torch is coupled to a 6-axis robotic arm from FANUC that moves with a constant linear speed of 0.05 mm·s^–1^ over the substrate. Silicon wafers (100) were used as substrates and were placed on a heating plate with a thermocouple fixed on the backside to measure their temperature. It is important to note that the thermocouple measurements were not precise, possibly due to a lack of good contact between the thermocouple and substrate. Thus, to measure the temperature, the plasma was directly directed onto the thermocouple, minimizing the potential heat losses that might occur due to air gaps or other factors. Besides, to enhance the thermal conductivity and ensure accurate readings, a copper foil was tightly wrapped around the thermocouple.

Optical emission spectroscopy (OES) measurements were carried out in the absence of a substrate to estimate the plasma gas temperature at multiple points (plasma torch exit, +10, +15, and +20 mm) and to describe the evolution of radiative species in the plasma afterglow region depending on the interaction with the precursor solution and the environment. OES measurements were carried out using a SpectraPro-2500i spectrometer (Princeton Instruments) and an optical fiber fitted with a collimator and connected to the spectrometer with a 50 mm focus (a stainless steel tube was used to guide and focus the measurements). A grating of 300 lines mm^–1^ blazed at 500 nm was used for its measurements.

An additional experiment was conducted under a nonoxidizing atmosphere, where the plasma torch was placed inside an acrylic box of 50 × 50 × 50 cm^3^ purged with N_2_, as shown in Figure S10. The latter was equipped with an O_2_ detector to measure the O_2_ concentration. Prior to deposition, N_2_ was purged to ensure a low O_2_ concentration. The test was performed when the O_2_ concentration was below 0.1%. No O_2_ was added to the N_2_ carrier gas, and no supplementary substrate heating was used. The test was conducted in the static mode. The temperature was measured by placing a thermocouple 15 mm from the plasma outlet.

### 2.2 Thin Film Characterization

The surface and bulk chemical compositions of the thin films were measured by XPS using a Kratos Axis instrument equipped with a monochromatic Al Kα X-ray source (*h*ν = 1486.6 eV). Energy calibration was performed based on adventitious carbon located at 284.8 eV. Three distinct measurements were performed: at the surface, after using argon clusters (Ar_1000_^+^) at 4 keV for 900 s to remove surface contamination, and after using Ar^+^ at 4 keV for 900 s to obtain the elementary composition of the bulk region. For comparison, the cobalt precursor (Co­(acac)_3_) powder was pressed onto an indium foil for characterization, and it was analyzed on the surface and after using argon clusters (Ar_150_^+^) for 300 s.

Identification of molecular groups present in the thin film was performed by static SIMS (TOFSIMS.5, IonTOF) equipped with a bismuth liquid metal ion gun for surface spectra acquisition. The dose density of Bi^3+^ primary ions was kept below 10^12^ ions·cm^–2^ bombardment to ensure static conditions.

The Raman spectra were recorded with a Renishaw inVia micro-Raman spectrometer at an excitation wavelength of 785 nm with a laser power of approximately 0.5 mW focused on a 5 μm^2^ spot. A Bruker D8 XRD with Cu Kα (λ = 0.154 nm) as the X-ray source was employed to study the structural properties of the films in a range of 30–70° with a step size of 0.02 and a grazing incident angle of 0.2°. The Williamson–Hall method was employed to calculate the lattice strain and crystallite size. The full width at half-maximum (FWHM) was deconvoluted for each peak using a pseudo-Voigt function, followed by subtraction from a reference sample (Al_2_O_3_ corundum), which was used for calculating instrumental broadening. Values are plotted as FWHM*cos­(θ) vs sin­(θ). Strain is obtained from the slope of the linear fitting, and the crystallite size is obtained from

$$D_{W-H}=\frac{K\lambda }{y_{\mathrm{intercept}}}$$

where *K* is the shape factor constant (0.94), λ is the X-ray wavelength of Cu Kα, and *y*_intercept_ is obtained from the linear fitting of the Williamson–Hall plot.

Scanning electron microscope (SEM) was employed to characterize the thickness and morphology of the coatings on a Hitachi SU-70 FE-SEM.

TEM lamella was prepared following the “lift-out” method with a FEI Helios Nanolab 650 focused ion beam scanning electron microscope (FIB-SEM). TEM analyses were performed on a JEOL JEM-F200 cold FEG microscope operating at an acceleration voltage of 200 kV. The crystalline nature of the specimen was analyzed by high-resolution TEM (HRTEM) imaging combined with fast Fourier transform (FFT) computation. Selected area electron diffraction (SAED) was performed to further confirm the crystalline phase identification. Electron energy loss spectroscopy (EELS) data were acquired under scanning-TEM mode (STEM), and energy loss spectra were acquired with the GATAN GIF Continuum ER post-column spectrometer. The convergence and collection angles were, respectively, 10.7 and 22.3 mrad, and the energy dispersion was 0.3 eV/ch.

For the electrochemical characterization of the coating, cyclic voltammetry (CV) and chronoamperometry (CA) measurements were performed with an Autolab PGSTAT302 potentiostat/galvanostat in a three-electrode configuration cell. The cell consisted of a Pt wire as the counter electrode, an Ag/AgCl (3 M KCl) electrode as the reference electrode, and a Co_3_O_4_ thin film deposited on fluorine-doped tin oxide (FTO)-coated glass as the working electrode. A 1 M potassium hydroxide (Sigma-Aldrich) solution with a pH of 13.6 was used as the alkaline electrolyte. All potentials were referenced to the reversible hydrogen electrode (RHE) using the Nernst equation *V*_RHE_ = *V*_Ag/AgCl_ + *V*_Ag/AgCl_^0^ + 0.0591 × pH. The current densities were normalized by using the geometric area of the electrode.

## 3 Results and Discussion

### 3.1 Synthesis of Crystalline Co_3_O_4_ Thin Films

Our strategy toward AP-PECVD of electrocatalytically active Co_3_O_4_ thin films relies on the use of a blown arc discharge, i.e., plasma torch, previously investigated for the atmospheric pressure and low-temperature deposition of other crystalline oxide thin films.^20,23^ Cobalt acetylacetonate (Co­(acac)_3_) was selected as the thin film precursor due to its stability at room temperature, its rather low onset volatilization temperature at atmospheric pressure (ca. 180 °C), and a bulk final decomposition temperature of 250 °C.^34,35^ For instance, Co­(acac)_3_ has been extensively used for the CVD and ALD of Co_3_O_4_ thin films.^13,14,36^ Co­(acac)_3_, a solid with limited vapor pressure at room temperature, was solubilized in various solvents to form a cobalt precursor solution with a suitable viscosity to allow its atomization as a fine mist and its transport to the plasma afterglow region (Figure 1). After several trials with different solvents (acetone, ethanol, isopropanol, and water) and different concentrations (not reported in this work for the sake of clarity), a 10 mM solution of Co­(acac)_3_ in acetone was selected. This choice was based on the high solubilization power of acetone, the low viscosity of the resulting cobalt precursor solution, and acetone’s low boiling point (56 °C), which enables its quick removal upon interaction with the blown arc discharge. Indeed, the plasma afterglow region is characterized by temperatures that range from 150 to 800 °C, depending on the input power and the distance from the exit of the blown arc discharge device, which is much higher than acetone’s boiling point. The cobalt precursor solution was injected into the plasma afterglow region at a distance (e) 1 mm above the substrate and a distance (d) 15 mm below the outlet of the plasma torch. Such distances (d) and (e), based on several preliminary trials, enable to prevent fast depletion of the cobalt precursor and the excessive formation of particles from uncontrolled gas-phase reactions.^20^ Considering the peak decomposition temperature of Co­(acac)_3_, the substrate was heated to 250 °C by an external plate heater. In addition, 40% O_2_ was added to the N_2_ carrier gas (in a total 2 L·min^–1^ gas flow) to ensure the full elimination of the organic moieties and to obtain the ideal stoichiometry of the Co_3_O_4_ thin film.^35,37^ Table S1 summarizes the parameters used during the AP-PECVD experiments reported in this work.

The thin film synthesized by AP-PECVD under open-air conditions (250 °C and 40% O_2_) exhibited a dark matte color without noticeable powder formation. Additionally, the thin film displays fair adhesion to the substrate as it was not degraded upon handling, cleaning, or sonication. The thin film thickness evaluated by SEM cross-sectional observation was between 300 and 500 nm (Figure S3a).

Raman spectroscopy analysis of the thin film synthesized by AP-PECVD under open-air conditions (250 °C and 40% O_2_) showed five well-defined peaks attributed to the Raman-active modes of Co_3_O_4_, i.e., A_1_g, Eg, and three F_2_g modes^10,38,39^ (Figure 2a, blue spectrum). Specifically, the most intense peak at 693 cm^–1^ is attributed to the A_1_g mode, which corresponds to the symmetric stretching of Co^3+^–O and the bending of Co^2+^–O, with the octahedral Co^3+^ being mainly responsible for the high intensity.^10,40,41^ The F_2_g mode at a low wavenumber (195 cm^–1^) corresponds to the complete translation of CoO_4_ (Co^2+^ on the tetrahedral site). The other assignments at 483, 522, and 621 cm^–1^ correspond to the vibrations of the tetrahedral and octahedral sites of Co_3_O_4_ ^10^. The low full width at half-maximum (FWHM) of the thin film indicates the formation of a highly crystalline oxide. Besides, the Raman spectrum of the thin film prepared by AP-PECVD under open-air conditions was compared to that of a Co_3_O_4_ reference sample fabricated from the oxidation of a metallic cobalt substrate under air at 600 °C for 24 h (Figure 2a, magenta spectrum). The two spectra present a good match with only a minor red shift of less than 2 cm^–1^ (Table S2). The peak shift in the Raman peaks could be related to stoichiometry variation or the presence of stress on the films. For instance, a shift to lower wavenumbers could indicate the presence of oxygen vacancies or tensile strain.^42^ The Cobalt-based thin film synthesized under nonoxidizing atmospheric conditions is discussed in detail in Section 3.3.3.

**Figure 2:**
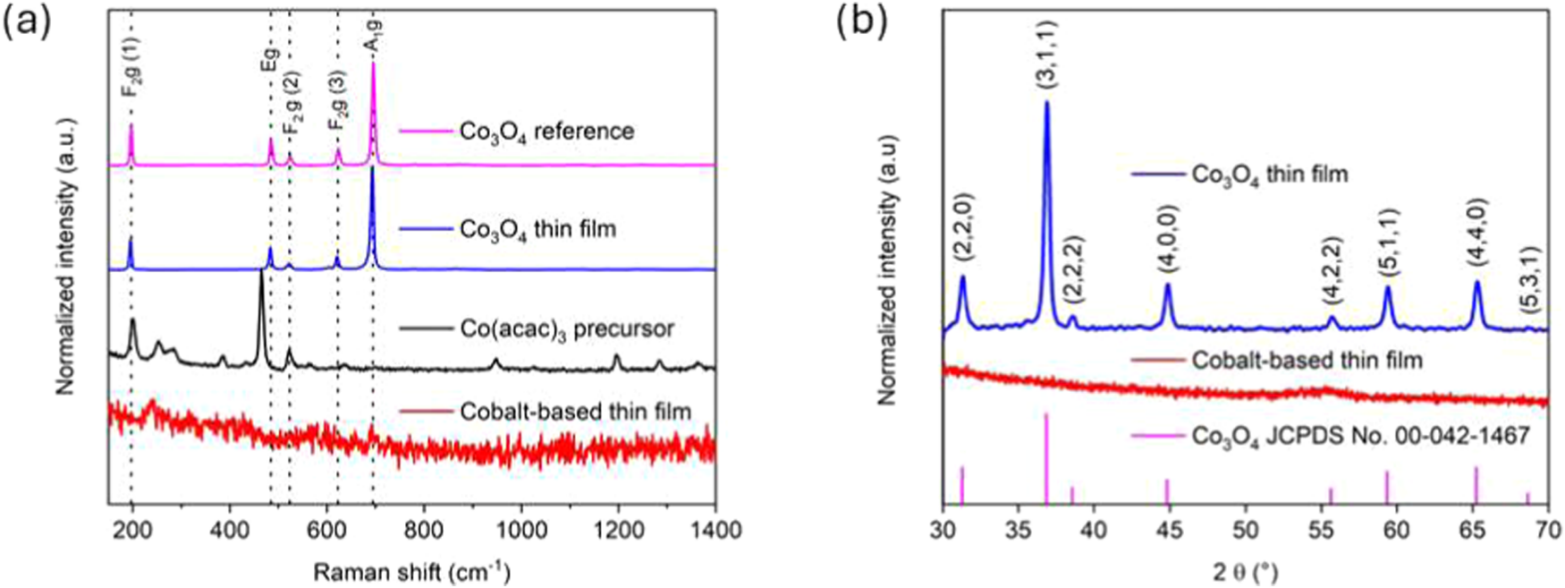
(a) Raman spectra of thin films prepared by AP-PECVD under open-air conditions at 250 °C and 40% O_2_ in a carrier gas (Co_3_O_4_ thin film, blue) and in a nonoxidizing atmosphere (cobalt-based thin film, red). The Raman spectra of the reference Co_3_O_4_ sample (Co_3_O_4_ reference, magenta), fabricated from the oxidation of a pure metallic cobalt sample, and the raw cobalt precursor (Co­(acac)_3_ precursor, black) are provided for comparison. (b) X-ray diffractograms of cobalt-based thin films prepared by AP-PECVD under open-air conditions at 250 °C and 40% O_2_ (Co_3_O_4_ thin film, blue) and under a nonoxidizing atmosphere (cobalt-based thin film, red). The Co_3_O_4_ reference (magenta) from the JCPDS database (no. 00-042-1467) is provided for comparison.

The X-ray diffractogram of the Co_3_O_4_ thin film prepared by AP-PECVD under open-air conditions (Figure 2b, blue spectrum) indicates that Co_3_O_4_ crystallizes in a cubic spinel-type structure in accordance with JCPDS card No. 00-042-1467. By comparison between the X-ray diffraction pattern and the JCPDS file, no preferential orientation is observed. Also, no peaks from other crystalline phases are noticeable. The crystallite size was calculated using the Williamson–Hall method, as explained in Section 2.2. The crystallite size was estimated to be ca. 41 nm (Figure S1c). Additionally, the Co_3_O_4_ lattice parameter *a* was calculated using the same approach as^37^

$$\sin^{2}\left(\theta \right)=\frac{\lambda^{2}}{{4a}^{2}}\left(h^{2}+k^{2}+l^{2}\right)$$

where θ is the Bragg angle, and *hkl* are the Miller indices.

The lattice parameter was calculated using a linear regression of the curve sin 2­(θ) vs *h* ^2^ + *k* ^2^ + *l* ^2^ (Figure S1), which gives the slope $A,\;\left(\frac{\lambda^{2}}{{4a}^{2}}\right)=0.009094$ . The calculated value of *a* is 8.078 Å, which is very close to the theoretical value for Co_3_O_4_ equal to 8.084 Å (JCPDS card No. 00-042-1467). This good agreement indicates only a slight lattice distortion and confirms the successful synthesis and deposition of a crystalline Co_3_O_4_ thin film from the AP-PECVD reaction of Co­(acac)_3_ under open-air conditions.

### 3.2 Electrocatalytic Properties of Co_3_O_4_ Thin Films

With the aim of evaluating the electrocatalytic activity toward the oxygen evolution reaction (OER) of the Co_3_O_4_ thin film reported above, fluorine-doped tin oxide (FTO)-coated glass was used as the substrate. The substrate was heated to 225 °C due to substrate restrictions, and 20% O_2_ was used to mimic the air environment. The resulting electrode was tested by cyclic voltammetry (CV) in an alkaline electrolyte (1 M KOH, pH 13.6; see Section 2 for further details). The bare FTO-coated glass was also measured under the same conditions as a reference, showing no electrocatalytic activity (Supporting Information, Figure S2). As depicted in Figure 3a (blue dashed line), the as-prepared Co_3_O_4_ thin film obtained by AP-PECVD under open-air conditions was active toward the OER, with an onset overpotential (η) of 390 mV to reach a 1 mA cm^–2^ current density. The onset overpotential of 390 mV required to achieve a 1 mA cm^–2^ current density is consistent with the values for undoped Co_3_O_4_ thin films in alkaline electrolytes, which typically range from 350 to 450 mV. The redox features observed at an *E*_1/2_ of ∼1.45 V vs RHE are assigned to Co^3+^ oxidation to Co^4+^ in the forward scan, and to Co^4+^/Co^3+^ reduction in the reverse scan.^6,7^ It is worth noting that the CoO_2_ phase (Co^4+^) is not stable under normal conditions. Subsequently, a chronoamperometry (CA) test was carried out in the same electrolyte by applying a constant potential of 1.65 V vs RHE for 2 h. As shown in Figure 3b, gas bubbles formed at the electrode surface, indicating the production of oxygen under anodic conditions. After CA (i.e., electrochemical aging), the Co_3_O_4_-based electrode was retested by CV (Figure 2a, light-blue straight line), showing an almost unaltered onset overpotential but increased current density at high potentials. Such an increase of the current density is likely related to the surface modification of the initial Co_3_O_4_ phase due to the formation of electrocatalytically active CoOOH species.^3,43,44^ In addition, electrochemical conditioning can remove the adsorbed species and reduce surface contamination through degradation or dissolution of organic moieties adsorbed on the surface, and initially block the active sites. Such observation correlates with the decreased Tafel slope for the Co_3_O_4_ thin film after CA, in comparison to the as-prepared Co_3_O_4_ thin film (Figure 3c), and a value (100.2 mV·dec^–1^) closer to the ones reported for other Co_3_O_4_ thin films.^10,45,46^ Chen et al. observed a Tafel slope of 101 mV·dec^–1^ for Co_3_O_4_ without vacancies. The value decreased to 72 mV·dec^–1^ as cationic vacancies were introduced into the material.^45^ Similarly, Xu et al. decreased the Tafel slope to 51 mV·dec^–1^ by increasing the Co^3+^/Co^2+^ ratio.^12^ Other strategies, such as the formation of nanoparticles, enhance the amount of catalyst active sites exposed to the electrolyte. Saddeler et al. reached ca. 50 mV·dec^–1^ for Co_3_O_4_.^47^

**Figure 3:**
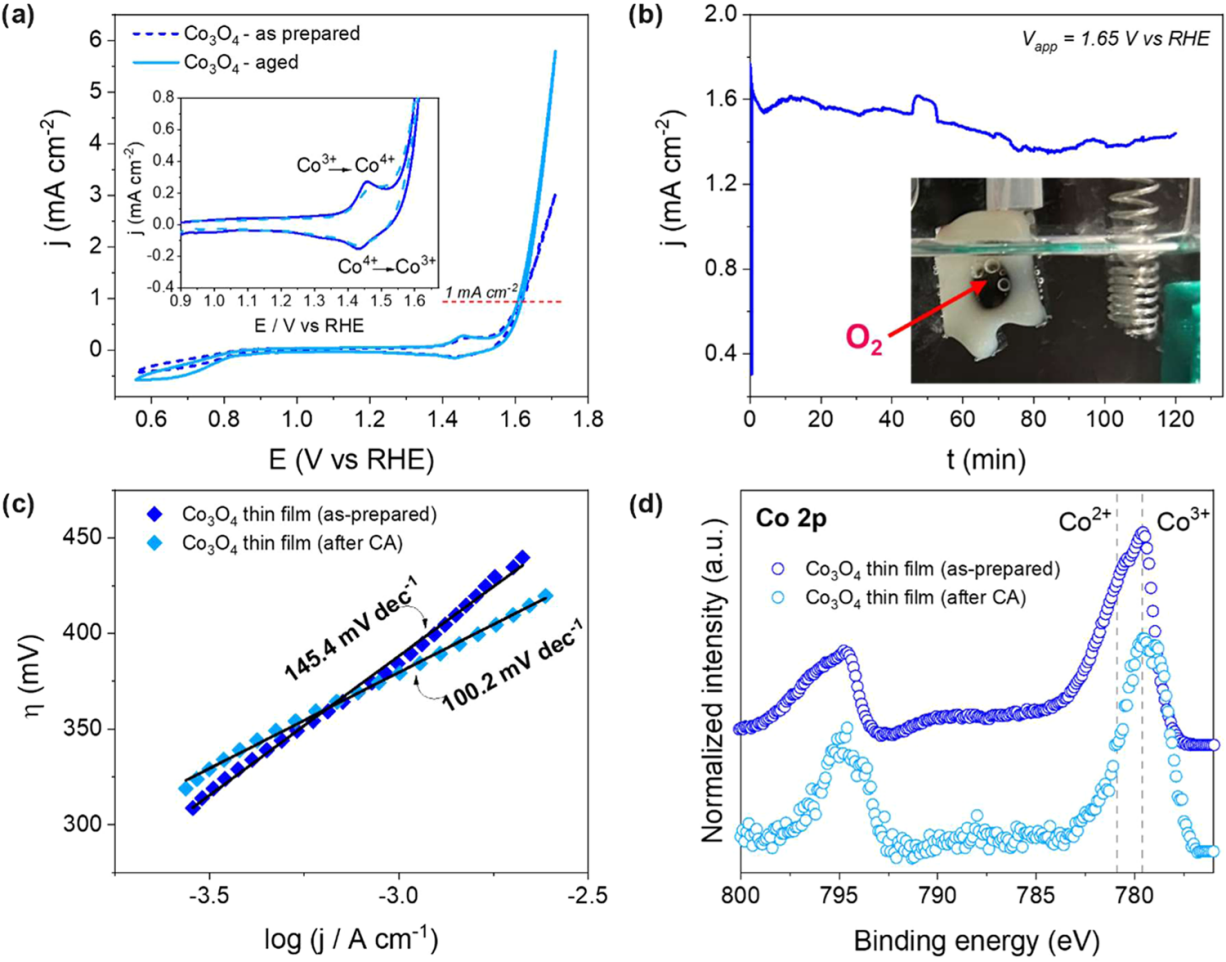
Electrocatalytic activity of the Co_3_O_4_ thin films deposited on fluorine-doped tin oxide (FTO)-coated glass by AP-PECVD under open-air conditions at 225 °C with 20% O_2_. (a) Cyclic voltammogram recorded at 100 mV s^–1^, in 1 M KOH, of the as-prepared Co_3_O_4_ thin film and after the chronoamperometry (CA) test. As shown in the inset, the zoomed-in region shows a redox feature corresponding to reversible Co^3+^ to Co^4+^ oxidation. (b) CA test carried out at 1.65 V vs RHE. The inset shows the formation of O_2_ bubbles at the surface of the Co_3_O_4_-based electrode. (c) Tafel plots of the as-prepared Co_3_O_4_ thin films and after the CA test. The calculated Tafel slopes are shown. (d) XPS spectra of the Co 2p core level of the as-prepared Co_3_O_4_ thin films and after CA. The reference binding energy positions of Co^2+^ and Co^3+^ are shown as a guide to the eye.

Further insights into the modification of the surface composition of the electrode due to electrochemical aging were obtained from X-ray photoelectron spectroscopy (XPS) analysis. Figure 3d shows the Co 2p core level signal, notably with the main Co 2p_3/2_ contribution at ca. 779.6 and 780.9 eV, characteristic of Co^3+^ and Co^2+^ species, respectively, in the as-prepared Co_3_O_4_ thin film. It is worth noting that the XPS analysis of materials containing transition metals, including cobalt, is often challenging due to the complexity of the 2p peak line shapes, featuring peak asymmetry, multiplet splitting, and the presence of shakeup satellites and plasmon loss structures.^48^ After electrochemical aging, a reduction in the Co 2p_3/2_ shoulder peak located at 780.9 eV and corresponding to Co^2+^ is observed (Figure 3d). This reduction is associated with an enhancement of the contribution related to Co^3+^ cations, supporting the conversion of the Co_3_O_4_ phase into a more electrocatalytically active CoOOH species at the electrode surface under OER operational conditions.^7,48,49^ Since ex situ XPS inherently captures the final state of the material rather than its precise in situ configuration under the OER conditions, the observed Co oxidation states and spectral features align well with the previously reported stable β-CoOOH phase that typically forms under moderate oxidation conditions in alkaline environments. This increased electrocatalytic activity confirms the observation of Strasser et al., which stated that Co^3+^ acts as a fast active site.^50^

The above observations confirm the potential application of the Co_3_O_4_ thin films prepared by AP-PECVD in catalysis technology for clean fuel production, such as hydrogen from water splitting, where the oxygen evolution reaction is a key step in the overall process. The following sections will focus on understanding the growth mechanisms enabling the formation of such a Co_3_O_4_ thin film and the impact of different parameters on the AP-PECVD process in the composition and microstructure of the thin films.

### 3.3 Nucleation and Thin Film Growth Mechanisms

To gain a deeper understanding of the Co_3_O_4_ thin film growth mechanism by AP-PECVD, the morphology and chemical composition were further examined. The elemental composition of the Co_3_O_4_ thin film was determined by XPS analysis at three different points: at the surface, after the elimination of adventitious carbon near the surface after 900 s of Ar_1000_^+^ cluster bombardment, and in the bulk region after 900 s of Ar^+^ bombardment (Figure 4). Satisfactorily, the measured carbon concentration in the bulk of the Co_3_O_4_ thin film prepared under open-air conditions was very low, reaching a mere 2 at. %. However, the surface of the as-prepared Co_3_O_4_ thin film initially exhibited high carbon concentrations (36 at. %) in the presence of organic carbon groups, indicating potentially significant adsorption of organic groups from fragments of the (acac) ligands and environmental contamination. A high carbon content (25 at. %) remained after elimination of the environmental contamination with the argon cluster sputtering. The presence of such a high level of carbon on the top surface may significantly hamper the electrochemical properties.

**Figure 4:**
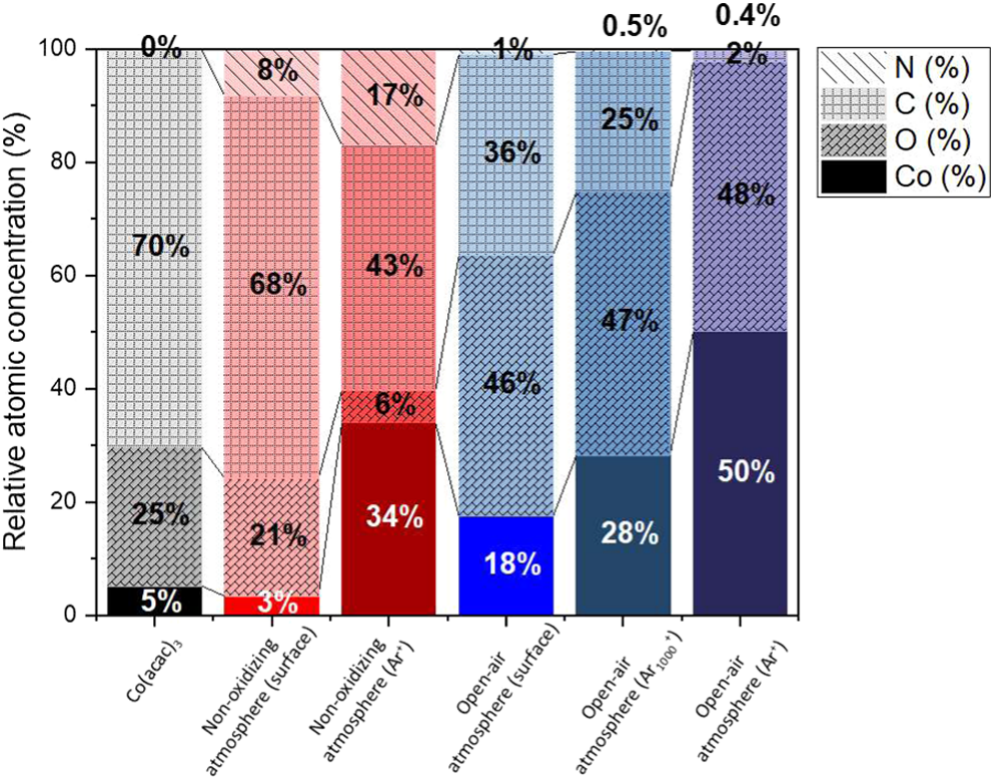
Relative atomic composition of Co_3_O_4_ thin films produced by AP-PECVD under open-air conditions (250 °C and 40% O_2_) and cobalt-based thin films produced by AP-PECVD under nonoxidizing atmospheric conditions. XPS measurements were performed on the surface of the samples after 900 s of Ar_1000_^+^ cluster sputtering and after 900 s of Ar^+^ sputtering.

The surface morphology of the Co_3_O_4_ thin films was investigated by SEM. Figure 5 presents top-view micrographs of the Co_3_O_4_ thin film prepared by AP-PECVD under open-air conditions. The grains exhibit a very fine grain size with angular facets. However, some regions show the presence of spheroidal grains and clusters of larger aggregates. According to previous reports, these aggregates likely originate from homogeneous reactions in the gas phase.^20^

**Figure 5:**
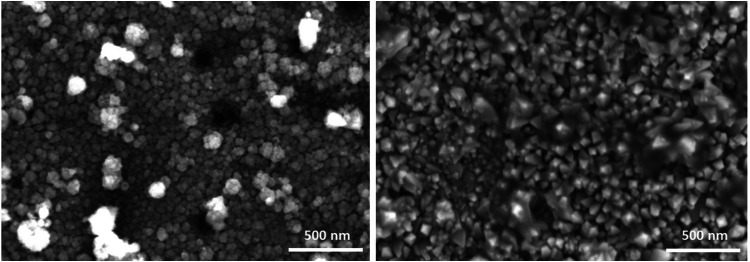
SEM top-view images of Co_3_O_4_ thin films produced by AP-PECVD under open-air conditions at 250 °C and 40% O_2_ addition to the carrier gas. The micrographs show two distinct regions of the Co_3_O_4_ thin film.

TEM analyses were carried out using an FIB-prepared lamella of the Co_3_O_4_ thin film prepared by AP-PECVD on a Si wafer under open-air conditions using a substrate heating temperature of 300 °C and a carrier gas composed of 40% O_2_. As shown in Figure 6a, Co_3_O_4_ has a dense and columnar structure with an average diameter of 30–100 nm (see orange arrow in Figure 6a). This value is in good agreement with the crystallite size determined previously by XRD. No delamination of the Co_3_O_4_ thin film from the substrate is observed, confirming its excellent adhesion to the Si substrate. Such a behavior, which is an asset of plasma processes, is promoted by the high-energy plasma species, ensuring surface activation.^29^ Selected area electron diffraction (SAED, shown in Figure S4b) and HRTEM imaging combined with FFT analysis were performed to investigate the crystalline nature of the Co_3_O_4_ thin film. Figure 6c,d show one of the well-ordered areas of the thin film and its FFT, respectively. This crystalline grain was identified as a Co_3_O_4_ cubic structure in the zone axis [110]. Indeed, the crystalline plane families are consistent with those already identified by XRD, thus confirming the crystalline structure of the Co_3_O_4_ thin film.

**Figure 6:**
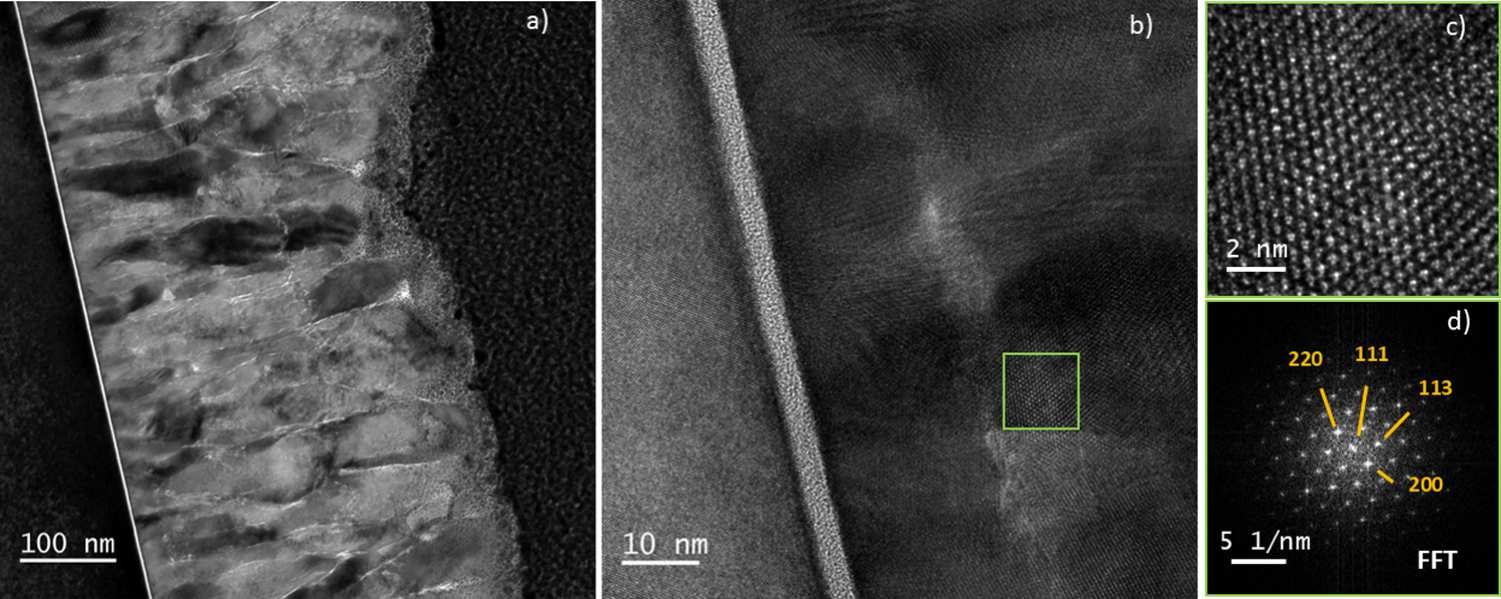
TEM observations of the Co_3_O_4_ thin film prepared from AP-PECVD of Co­(acac)_3_ under open-air conditions, a substrate heating temperature of 300 °C, and a gas mixture with 40% O_2_ for cobalt precursor injection: (a) Bright-field TEM image of the Co_3_O_4_ thin film cross-section. (b) HRTEM micrograph of the thin film at the interface with the silicon substrate. (c) Enlarged image cropped from image b (within green solid squares). (d) Fast Fourier transform (FFT) of images (c).

The high-angle annular dark field (HAADF) STEM image (Figure S4c) shows darker regions (yellow circles), which may correspond to lighter elements compared to Co and O, or to voids. Using electron energy loss spectroscopy (EELS), the presence of carbon was confirmed (Figure S5). Carbon forms a mixed phase with cobalt and oxygen, possibly coming from the incompletely fragmented Co­(acac)_3_ precursor. XPS measurements indicated the presence of carbon in the bulk, and Figures S4 and S5 confirm its distribution in the thin film. The darker regions are heterogeneously dispersed, suggesting that these carbonaceous regions were trapped during the Co_3_O_4_ thin film growth.

Section 3.1 showed that a highly crystalline Co_3_O_4_ thin film with a low level of carbon impurities in the bulk and interesting electrocatalytic activity was formed using the investigated AP-PECVD setup. However, carbon residues were observed closer to the surface (Figure 4 “open-air” and “open-air (Ar_1000_^+^)”) and, in minor quantities, in the bulk (Figure 4 “open-air (Ar^+^)” and Figure S5) of the Co_3_O_4_ thin films. Thus, to better understand the conditions responsible for AP-PECVD of crystalline and low-carbon Co_3_O_4_ thin films, we evaluated the significance of several process parameters, including heating and O_2_ content in the carrier gas and surrounding atmosphere.

#### 3.3.1 Influence of O_2_ Concentration in the Carrier Gas

A complementary series of thin films was prepared by varying the O_2_ fraction in the N_2_ carrier gas (from 0 to 60%) while keeping the total carrier gas flow at 2 L·min^–1^ (which represents 0–2.2% O_2_ from the total gas flow comprising the plasma, carrier, and atomizing gases). The substrate heating temperature was kept at 250 °C for all of the O_2_ concentrations investigated in this section. The addition of O_2_ was, initially, considered critical to produce low carbon–nitrogen coatings and to ensure proper stoichiometry, such as for other produced thin films.^25^ The initial tests, reported in Section 3.1, were conducted with 40% O_2_ in the carrier gas, which yielded low carbon contamination in the bulk region but not in the surface region of the Co_3_O_4_ thin film.

Since the carbon contamination was higher near the surface, the chemical composition of this region of the Co_3_O_4_ thin film was carefully measured by XPS, followed by depth-profiling ToF-SIMS. Additionally, Raman spectroscopy was used to determine the production of the cobalt oxide phase as a function of O_2_ concentration in the carrier N_2_ gas (from 0% to 60% O_2_). Regardless of the oxygen amount, the same 5 Raman peaks, as shown in Figure S6, were observed, all corresponding to Co_3_O_4_. Besides, the full width at half-maximum (FWHM) is very similar in all cases, indicating no evident influence on crystallinity.^51^ Consequently, the addition of O_2_ to the carrier gas does not significantly influence the formation of the Co_3_O_4_ phase in the AP-PECVD setup used in this work.

XPS analysis revealed a negligible impact of O_2_ concentration in the carrier gas on the carbon concentration in the thin films prepared under open-air conditions (Figure S7a). Indeed, the small variation in carbon concentration, from 23.4% to 24.8%, is comprised in the error margin. Furthermore, irrespective of the O_2_ concentration in the carrier gas, negligible amounts of N_2_ (<1%) were observed in the bulk region (Figure S7a). The Co_3_O_4_ thin film prepared under open-air conditions without the addition of O_2_ to the carrier gas was further analyzed using ToF-SIMS (Figure 7a). The depth profile revealed a constant intensity for cobalt and oxygen ions through the thin film and the presence of carbon-related ions (CN^+^, CO^+^) near the surfaces. Interestingly, the concentration of carbon-related ions dropped rapidly at the beginning of the sputtering process (0 to 5000 s) and then roughly stabilized at low intensities deeper into the bulk. These observations align well with the XPS data from the bulk region of the sample with 40% O_2_ (Figure 4), indicating that carbon groups are mainly present close to the surface, possibly coming from fragments of the precursor and postprocessing environmental contamination.

**Figure 7:**
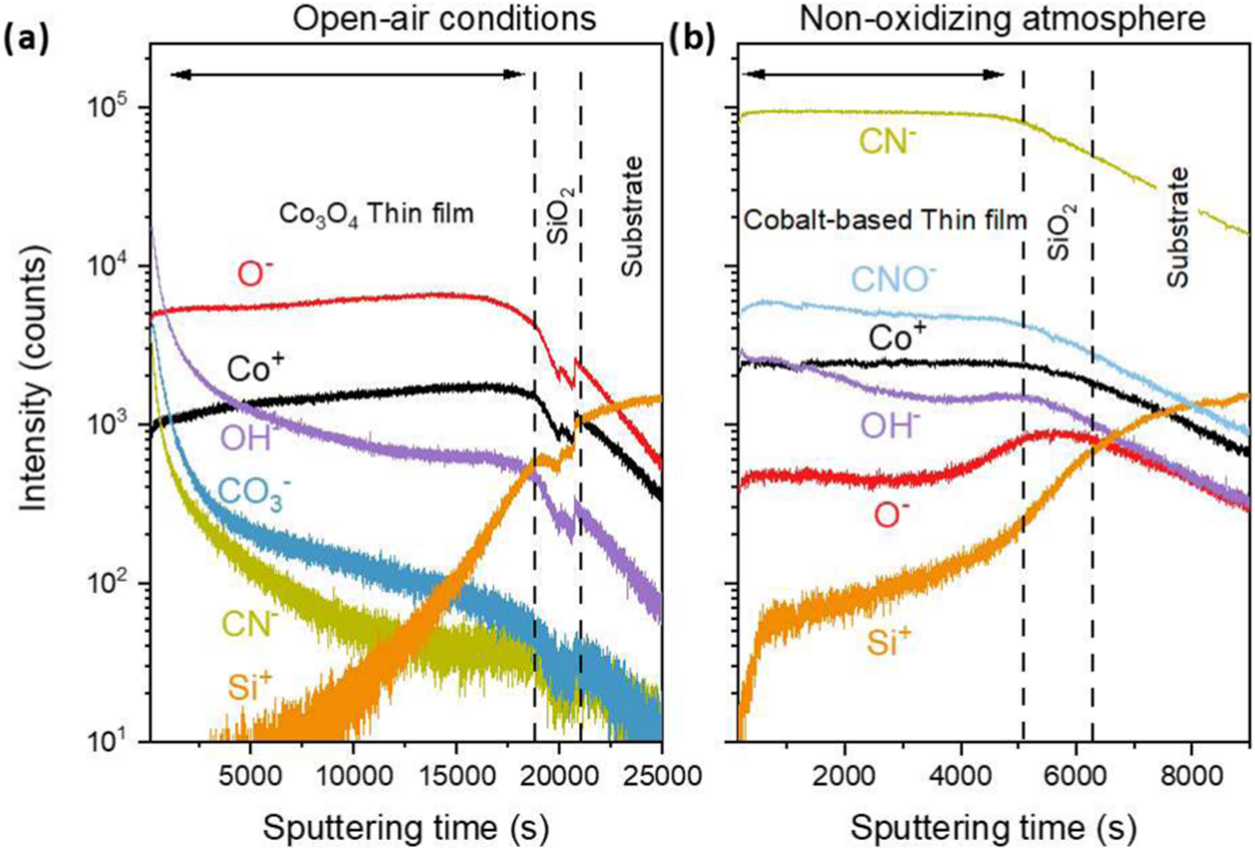
ToF-SIMS profile of the thin films prepared by AP-PECVD under open-air conditions at 250 °C (Co_3_O_4_ thin film in (a)) and under a nonoxidizing atmosphere (cobalt-based thin film in (b)) without the addition of O_2_ in the carrier gas. The thin film/SiO_2_ native layer interface was identified by the variation of the inflection point from the different elements observed in the profile.

Therefore, in conclusion, TOF-SIMS profiles and XPS analyses indicate that the addition of O_2_ to the carrier gas does not significantly impact the removal of organic ligands and residual contaminants. Similarly, Raman analyses suggest no variation in the crystallinity of the Co_3_O_4_ thin films.

#### 3.3.2 Influence of Substrate Temperature

The influence of the substrate heating temperature (no external heating, 200 °C, 250 °C, as described in Sections 3.1 and 3.2, and 300 °C) on the formation of Co_3_O_4_ thin films and their carbon contamination was investigated. For this set of experiments, the carrier gas concentration was set to 40% O_2_. Regarding the thin film composition, even without external heating, a fast carbon decrease from the surface to the bulk region (ToF-SIMS Figure S9) was observed, suggesting that the plasma–precursor interactions were sufficient to remove the precursor’s organic ligands. However, the surface contamination seems to be lower at 300 °C. XPS measurements were performed at the near-surface region, and a decreased contamination was identified at 300 °C, while at lower temperatures, the results remained close, considering the relative errors (Figure S7b). The influence of different substrate heating temperatures was further analyzed by Raman spectroscopy and XRD. Irrespective of the substrate heating temperature, the Raman spectra of the thin films exhibit five characteristic peaks of Co_3_O_4_ with a minimal shift between each other (less than 1 cm^–1^), showing good agreement with the reference Co_3_O_4_ sample prepared at 600 °C (Figure S8). Additionally, as performed by Wu et al., the $\frac{F_{2}g\left(1\right)}{A_{1}g}$ intensity ratio for all conditions was compared to detect slight variations in the structure of the thin films.^41^ The F_2_g peak refers to tetrahedral sites corresponding to Co^2+^, and the A_1_g peak has mainly contributions of the octahedral sites, corresponding to Co^3+^. The FWHM of the A_1_g peak slightly reduces as the substrate’s heating temperature increases (Table S2), suggesting enhanced crystallite growth at higher substrate temperatures. Additionally, the $\frac{F_{2}g\left(1\right)}{A_{1}g}$ ratio was observed to decrease as the substrate heating temperature increased, suggesting a higher presence of cobalt in the octahedral sites (Co^3+^) for the thin films produced at higher substrate heating temperatures.

XRD analyses performed for the thin film synthesized with no external heating or at 250 and 300 °C suggested no preferential orientation, with peak intensities matching the JCPDS card No. 00-042-1467 for Co_3_O_4_ with a cubic spinel structure. Moreover, a decrease of the lattice parameter (Figure S1) was observed from 8.080 to 8.078 and 8.074 Å for no substrate heating to 250 and 300 °C, respectively. The decrease in the lattice parameter with increasing substrate heating temperature correlates well with previous Raman observations that indicated an increase in Co^3+^ in the lattice. Since the ionic radius of Co^3+^ is smaller than that of Co^2+^, a decrease in the lattice parameter is expected.

Hence, substrate heating has a slight influence on the AP-PECVD formation of crystalline Co_3_O_4_ thin films, and only minor changes were observed, such as an increase in Co^3+^ cations and a decrease in surface contamination. Therefore, the heat and energy required to crystallize the Co_3_O_4_ thin film likely originate from the plasma and its highly energetic species.

To characterize the plasma gas temperature and plasma chemical composition, OES analysis was performed in the absence of a substrate at different points of the plasma afterglow. The O_2_ concentration in the carrier gas used to transport the precursor solution was maintained at 40%. Figure 8a shows the radiative transitions measured at three different positions in the plasma afterglow (0, 10, and 15 mm from the exit of the plasma torch). Three main contributions are observed: the transitions of the N_2_ second positive system, the NO β system, and the N_2_^+^ first negative system.^52^ The gas rotational temperatures at different distances were evaluated using the LIFBASE software.^53^ The recorded emission spectra of the NO β system and the N_2_^+^ first negative system were superposed on the simulated spectra for these systems. To obtain a rotational temperature value with greater accuracy, the calculation simulated the spectra from 280 to 400 nm. It is common to consider, at atmospheric pressure, that this rotational temperature corresponds to the temperature of the gas in the plasma afterglow,^54,55^ although the plasma afterglow contains species with a much higher energy.

**Figure 8:**
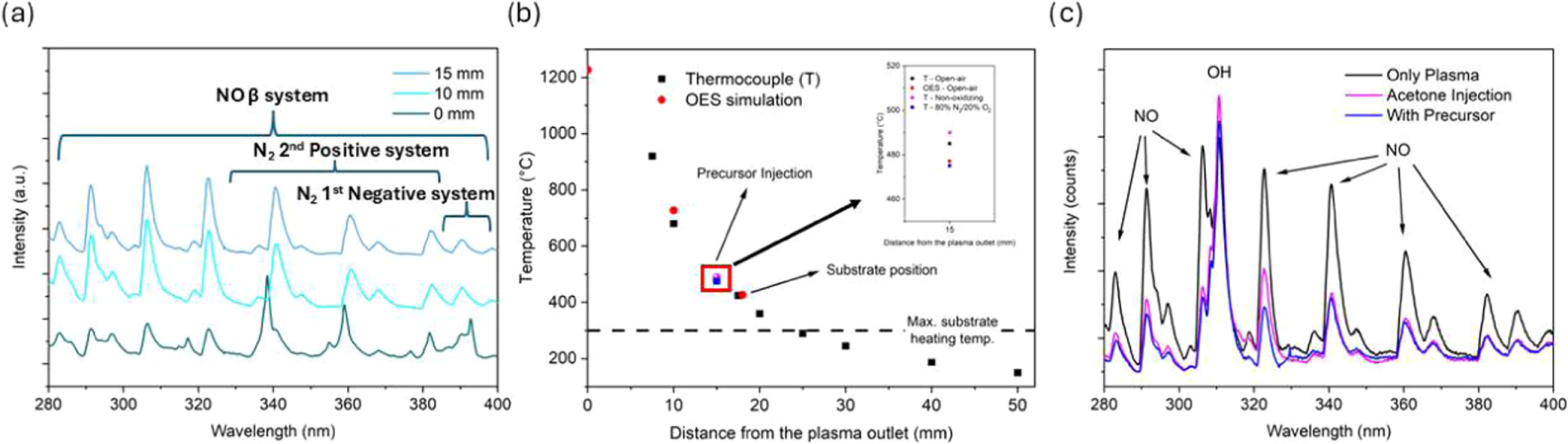
OES measurements were carried out in the absence of a heating plate and using 40% O_2_ in a carrier gas. (a) Evolution of plasma chemistry as a function of distance from the plasma torch outlet. While close to the outlet (0 mm), multiple species appear (N_2_, N_2_^+^, and NO), and only NO was observed when moving away from the outlet. (b) Temperature profile based on OES measurements at different points of the plasma afterglow (red circles) and thermocouple measurements at different distances (black squares). The temperature decreases rapidly as the measurement is performed further away from the plasma outlet. Thermocouple (T) measurements are also shown in a controlled atmosphere (nonoxidizing, magenta spheres) and (80% N_2_/20% O_2_, blue squares). (c) OES measurements with only plasma (black), plasma and acetone (magenta), and plasma + solution (blue).

Close to the plasma torch exit (0 mm), the plasma gas temperature is estimated to be 1150 °C and decreases quickly to 750 °C at 10 mm. The temperature further reduces to 480 °C at 15 mm (precursor injection) from the outlet and to 425 °C at 17.5 mm (substrate position). To confirm the influence of gas temperature on thin film production, these values were independently verified using thermocouple measurements. Both methods confirm that a high temperature (>400 °C) was reached when the plasma was over the sample (Figure 8b), although it decreases as it moves away. The cobalt precursor injection was set at 15 mm from the plasma torch exit, and the substrate position at 17.5 mm, and the thermal gradient between the injection point and the substrate was considered relatively small.

Therefore, the plasma gas temperature at the position of the precursor injection, about 480 °C, is well above the decomposition temperature of the Co­(acac)_3_ precursor (250 °C)^34,35^ and explains the lack of major changes in crystallinity for the highest substrate heating temperature (300 °C) since it is well below the plasma gas temperature. Despite the high plasma gas temperature, thin film formation is expected to occur at the substrate’s surface. The residence time of the cobalt precursor in the plasma afterglow is extremely short due to the high gas speed (>200 m·s^–1^),^55^ and the proximity of the cobalt precursor injection outlet and substrate. As a result, the deposition process is mainly driven by heterogeneous reaction mechanisms at the surface of the substrate, yielding the formation of dense thin films, as can be seen in the TEM images (Figure 6a) and in SEM top-view images (Figure 5), indicating the presence of only a few regions with large particle formation.

Thus, external substrate heating is not crucial when using the investigated plasma torch. Schubert et al. demonstrated that by providing high temperature and oxygen, Co­(acac)_3_ produces Co_3_O_4_ alongside byproducts at 230 °C. They stated that the decomposition process relates to that of Cu acetate, which occurs by the formation of radicals.^35^ In the present work, the plasma reactive species likely accelerate the radical formation and, therefore, the Co_3_O_4_ thin film formation.

#### 3.3.3 Influence of O_2_ in the Gas Environment

Figure 8a depicts the rapid variation in plasma chemistry along the plasma afterglow. While N_2_ and N_2_^+^ are the main species observed immediately at the plasma torch outlet, NO becomes predominant 10 mm below. N_2_^+^ species are highly energetic and promptly react with oxygen-bearing molecules present in the air atmosphere, producing NO in blown arc discharges.^52,54^ Consequently, N_2_^+^ species have a short lifetime^56^ and do not likely participate in the AP-PECVD reaction of (Co­(acac)_3_). Figure 8c shows the OES spectra acquired at the substrate position immediately below the injection system. Measurements were performed with only plasma (black), plasma and acetone (magenta), and plasma and solution (blue). The graph shows the presence of two main species, NO and OH. The latter is a common species in atmospheric pressure plasmas, resulting from the fragmentation of water molecules present in air. Since OH species were not observed in the other measurements (Figure 8a), they are assumed to be mainly produced in the region surrounding the plasma afterglow, while other reactive oxygen–nitrogen species (RONS) are mainly observed in the central region of the plasma afterglow. The presence of RONS indicates the effective mixing of plasma and the surrounding air environment. RONS and metastables are the main species present in the plasma afterglows. Electrons, ions, and other excited species have lower lifetimes, tending to decay by deexcitation or through collisions with other molecules.^57^ The OES measurements clearly illustrate modifications of the plasma gas composition with the addition of the solvent and precursor solution. There is a decrease in the intensity of the NO peaks, while that of the OH peak increases. The decreased intensity of the NO species points to a quenching mechanism, suggesting energy transfer from NO to the injected molecules. The OH increase possibly results from H abstraction from the organic groups.^58^ RONS have long lifetimes and are expected to be active during the plasma–precursor interaction at the substrate surface.^59^ Similarly, metastable molecules possessing long lifetimes, such as N_2_ metastables (N_2_^m^), should also be present^29,60^ despite their absence of detection by OES measurements,^61,62^ and may contribute to thin film production, especially by transferring their energy to the precursor. The presence of excited oxygen-rich radicals under open-air conditions possibly explains the lack of difference observed when varying the O_2_ concentration in the carrier gas. While the O_2_ in the carrier gas is injected under the same conditions as the precursor solution, at room temperature, the oxygen-rich radicals, drawn from the surrounding open-air atmosphere, already possess higher energies when encountering the precursor.

Plasma chemistry is very complex, and thin film formation most likely relies on the synergistic effect of plasma reactive species and convective heat from the plasma gas.^63^ In the present work, the plasma gas temperature is relatively high and likely participates in thin film formation. Nevertheless, radicals (i.e., NO, OH, etc.) resulting from the interactions between the plasma and open-air environment likely contribute to the thin film growth and composition. Hence, we used reactions R1–R3 to illustrate a possible mechanism for thin film formation, whereas other possible reactions may exist. This hypothesis considers that metastable molecules and RONS participate in thin film formation via three main reactions. First, precursor radicals (precursor activation)^64^ are formed through the fragmentation of weak precursor bonds^29^ (R1) or through interaction with NO species (R2). Precursor activation is suggested to occur in the gas phase through the formation of radicals, also known as film-forming species. Although R1 is suggested to occur through the impact of N_2_ metastables, another pathway is dissociation by electron impact. However, the latter was not considered in this work since the electron density in the afterglow is expected to be very low.^57,65^ As suggested in Figure 8c, the NO species possibly participate in the activation process. NO species have high internal energies (5.7 eV),^66^ enabling them to fragment a wide variety of organic molecules.

R1 $${\mathrm{Co}‐\mathrm{acac}}_{3}+N_{2}^{m}\to {\mathrm{Co}‐\mathrm{acac}}_{\left(\mathrm{ligand}\right)}^{•}+{\mathrm{acac}}_{\left(\mathrm{ligand}\right)}^{•}+N_{2},\mathrm{NO}\cdot \cdot \cdot$$

R2 $${\mathrm{Co}‐\mathrm{acac}}_{3}+{\mathrm{NO}}^{•}\to {\mathrm{Co}‐\mathrm{acac}}_{\left(\mathrm{ligand}\right)}^{•}+{\mathrm{acac}}_{\left(\mathrm{ligand}\right)}^{•}+N_{2},\mathrm{OH},\mathrm{NH}\cdot \cdot \cdot$$

Following the formation of precursor radicals, they could either react between them^64^ or through potential chemical reactions^67^ with oxygen-bearing molecules, such as NO^•^ and OH^•^. R3 will deplete the acetylacetonate organic ligands (O_2_C_5_H_7_) and provide the oxygen atoms necessary for the oxidation of cobalt species and the formation of Co_3_O_4_. The elimination of carbonaceous species^68^ and the formation of oxide (R3) likely occur at the surface. While the former was suggested by Reuter et al.^68^ when investigating SiO_*x*_ formation, the latter is indicated by SEM and TEM images, which suggest that homogeneous reactions are not the main contributor to thin film growth.

$${\mathrm{Co}‐\mathrm{acac}}_{\left(\mathrm{ligand}\right)}^{•}+{\mathrm{NO}}^{•},{\mathrm{OH}}^{•}\overset{\mathrm{thermal}\;\mathrm{energy}}{\to }{{\mathrm{Co}}_{3}O}_{4}+{\mathrm{CO}}_{x},{\mathrm{CH}}_{x},{\mathrm{NH}}_{x},\mathrm{HCN}\cdot \cdot \cdot$$ R3

This assumed mechanism of Co_3_O_4_ thin film synthesis requires the formation of RONS prior to their interaction with the precursor solution mist to form Co_3_O_4_ thin films. To prove the necessity of RONS formation in the plasma phase, the AP-PECVD experiment was conducted in a nonoxidizing atmosphere using N_2_ gas. The O_2_ measurement in the box showed that the O_2_ concentration was below 0.1%. No O_2_ was added to the N_2_ carrier gas, and no supplementary substrate heating was used. Temperature measurements (Figure 8b) indicate a very low variation in temperature (<20 °C) at 15 mm from the plasma outlet between nonoxidizing, open-air conditions and synthetic air (80% N_2_/20% O_2_). The color of the plasma changed from yellow-blue under open-air conditions to violet (Figure S11) under nonoxidizing atmospheric conditions, which is typical of N_2_ plasma emission. In this experiment, the only expected source of oxygen is therefore cobalt precursors (Co­(acac)_3_), which have 6 oxygen atoms surrounding the cobalt metal center, and acetone (C_3_H_6_O), used as the solvent. In comparison to open-air conditions, the oxygen supply is predicted to be limited. Consequently, the absence of O_2_ prevents a sufficient oxygen supply for oxide film formation.

After 10 min of static experiment in a nonoxidizing atmosphere, the AP-PECVD reaction of Co­(acac)_3_ yielded an adherent black coating on the silicon substrate. SEM cross-sectional observation indicates a thin film thickness of ca. 120 nm, which indicates a drastic decrease in the thin film growth rate under nonoxidizing atmosphere (85% lower growth rate than under open-air conditions). Moreover, the X-ray diffractogram (Figure 2b) and Raman spectrum (Figure 2a) of the cobalt-based thin film produced under a nonoxidizing atmosphere do not exhibit any obvious peaks, in contrast to those obtained for the Co_3_O_4_ thin film formed in air (Figure 2).

Analyses of the thin film prepared by AP-PECVD under open-air conditions and under a nonoxidizing atmosphere were carried out by ToF-SIMS depth profiling (Figure 7) and XPS (Figures 4 and 9), and the obtained results were compared. The XPS survey spectra of the thin films formed in an open-air atmosphere (blue) and nonoxidizing atmosphere (red) in the bulk region are compared with the spectrum of the cobalt precursor (black). The XPS survey spectrum of the cobalt-based thin film prepared under a nonoxidizing atmosphere highlights the presence of carbon and nitrogen, while under open air, mainly cobalt and oxygen were observed. Moreover, more contamination of the thin film by carbonaceous species is observed for the thin film synthesized under nonoxidizing conditions than in an open-air environment. Indeed, whereas the carbon concentration in the bulk of the thin film formed in open air is very low, about 2 at. %, it is very high in the thin film formed in a nonoxidizing atmosphere, about 43 at. % (Figure 4). The bulk of the thin film formed in a nonoxidizing environment also contains a high concentration of nitrogen, about 17.0 at. %. ToF-SIMS analyses reveal contamination compounds with much higher intensity in the thin film formed in a nonoxidizing atmosphere than in an open-air atmosphere: CO_3_^–^ and OH^–^, which are very likely residues from the acac rings, and CNO-, which is very likely produced from the interaction of the plasma species (e.g., N_2_ metastables) and the precursor’s ligand. The latter is also possibly the pathway for the incorporation of CN- ions, which is consistently observed throughout the cobalt-based thin film depth.

**Figure 9:**
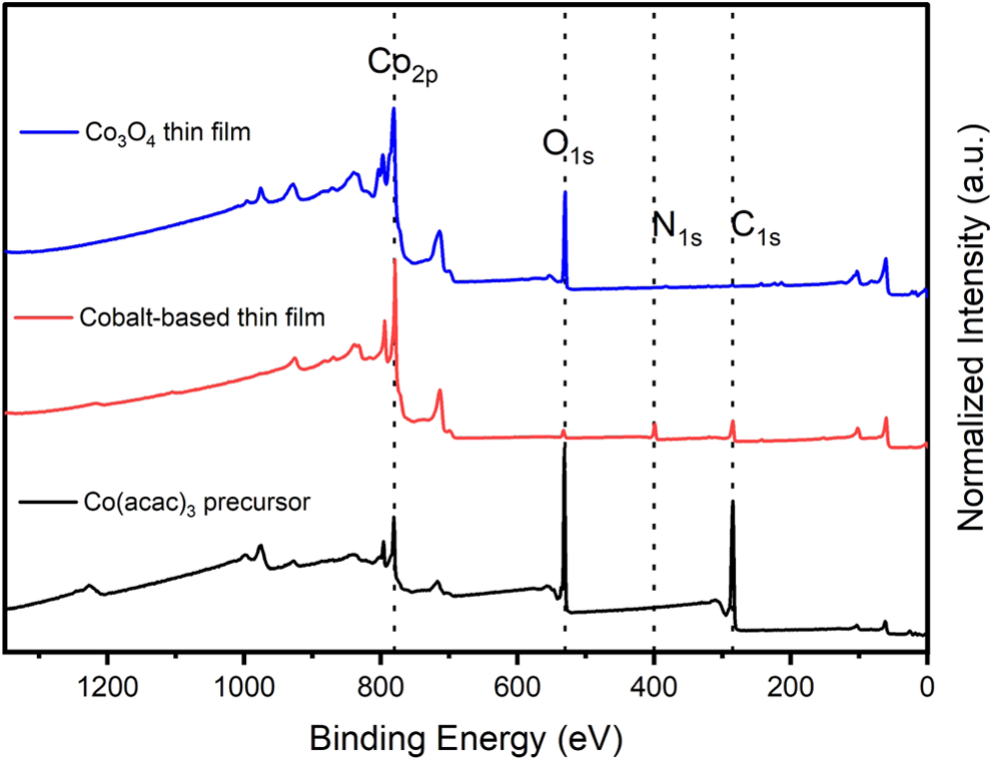
XPS survey spectra of the cobalt precursor (Co­(acac)_3_) (black), cobalt-based thin film produced by AP-PECVD under a nonoxidizing atmosphere (red), and Co_3_O_4_ thin film produced by AP-PECVD under open-air conditions at 250 °C and 40% O_2_ in a carrier gas (blue). The survey spectra correspond to the thin films after 900 s of Ar^+^ sputtering for the elimination of adventitious carbon and correct analysis of the bulk film. The Co­(acac)_3_ precursor spectrum corresponds to the measurement after 300s of Ar_150_^+^ sputtering for the elimination of adsorbed organic groups.

Thus, the lack of O_2_ in the environment reduces the elimination of impurities, mainly carbon, and allows for the incorporation of nitrogen. A lower O_2_ concentration likely leads to less quenching of the highly energetic N_2_ species. Excited N_2_ species and N atoms are considered responsible for nitrogen adsorption on the surface.^69^ The high remaining carbon content indicates that the interaction with oxygen-rich compounds is critical to volatilize carbon species, i.e., CO_*x*_. The N_2_ species do not promote such reactions. However, NO radicals, the major component under open-air conditions, and their parent species have very high oxidizing potential.^58^ The thin film formed under nonoxidizing atmospheric conditions contains cobalt but very low oxygen, which differs from the thin film formed in open air. The Co^+^ and O^–^ intensities observed by ToF-SIMS (Figure 6) confirm the reduced oxygen concentration along the thin films produced in a nonoxidizing atmosphere compared to an open-air atmosphere. Similarly, the Co/O atomic ratio measured by XPS (Figure 4) emphasizes this difference. While the ratio is about 6 for the thin film formed in a nonoxidizing atmosphere, the value is close to unity for the Co_3_O_4_ thin film formed in an open-air atmosphere. The high Co/O ratio value highlights the fact that cobalt is present in a phase other than an oxide. Interestingly, this value is much lower for the precursor (1/5), which suggests that the plasma species formed under a nonoxidizing atmosphere break the Co–O bonds of Co­(acac)_3_. The latter likely occurs, as suggested in R1 and R2, where one acac ligand dissociates from the initial structure, generating radicals. Previous works^25–70^ have evaluated the bond dissociation energy (BDE) of the cobalt–oxygen bond to a value close to that of C–O (ca. 3.8 eV), which is much lower than that of C=O (ca. 7.8 eV) and also present in the acac rings. Consequently, the Co–O bond is expected to be more easily depleted, with the oxygen remaining partly attached to the carbon.

A similar reasoning can be used for the Co/C ratio. The Co/C ratio also increases in the thin films produced under a nonoxidizing atmosphere (0.78) and under open-air (25) conditions compared to the Co­(acac)_3_ precursor (0.07). Under open-air conditions, carbon depletion possibly follows the previously suggested mechanisms (R1–R3), indicating further fragmentation of the detached ligand into CO_2_ and carbon-rich moieties that can further react with RONS. Under a nonoxidizing atmosphere, the mechanism varies. N_2_ and its excited species possibly fragment the precursor, as illustrated in R1, reducing the levels of oxygen and carbon. Additionally, these species are likely to interact with the precursor radicals to further reduce carbon and oxygen. The latter should occur by producing volatile species, but can also be observed by the incorporation of CN^–^ and CNO^–^ ligands in the thin film.

## 4 Conclusions

Crystalline and electrocatalytically active Co_3_O_4_ thin films were successfully synthesized under open-air conditions using AP-PECVD with Co­(acac)_3_ as the precursor. The influence of various process parameters on the composition, crystallinity, and quality of AP-PECVD thin films was studied. The main results are as follows:

- Thin films deposited on FTO samples showed promising catalytic potential, similar to Co_3_O_4_. Their performance increased after one scan, possibly due to the elimination of organic groups adsorbed to the surface and the conversion of Co_3_O_4_ to β-CoOOH. Besides, by increasing the substrate heating temperature, it is possible to tune the Co^3+^/Co^2+^ ratio, which is considered a pathway for increasing the catalytic potential.
- No major influence of the substrate temperature by an external heating source, up to 300 °C, was observed. This observation is explained by the high temperature already promoted by the plasma afterglow on the substrate surface.
- The presence of O_2_ from the surrounding open-air conditions was demonstrated to be necessary to form stoichiometric Co_3_O_4_ thin films and eliminate residual impurities, such as carbon and nitrogen. Oxygen atoms from cobalt precursors or solvents are not sufficient to form Co_3_O_4_. This conclusion is supported by (i) the AP-PECVD experiments performed in a nonoxidizing atmosphere, therefore containing a very low concentration of oxygen-bearing molecules (O_2_, H_2_O), and (ii) the series of AP-PECVD experiments performed for varying the O_2_ concentration in the carrier gas. The latter indicated no clear variation in the produced phase or in the chemical composition of the thin films. The lack of significant importance of O_2_ in the carrier gas possibly arises from the fact that O_2_ represents a minimal concentration compared to the total gas flow, while up to 20% O_2_ concentration can be drawn from the open-air environment. Additionally, the O_2_ from the open air was already excited before encountering the precursor, while O_2_ from the carrier gas was excited under the same conditions as the precursor.
- The formation of Co_3_O_4_ is assumed to occur through radical formation, where metastables and other reactive species initially fragment the cobalt precursor, producing radicals, which then react with the oxygen-rich species formed in the plasma by the reaction with the oxygen-bearing molecules contained in the surrounding air atmosphere.
- Co_3_O_4_ thin films are obtained using a solvent-free, open-air, and potentially scalable method, which enhances their appeal for sustainable energy conversion applications. Furthermore, one of the major advantages of the AP-PECVD process is its suitability for the growth of doped thin films from the simultaneous injection of multiple metal precursors, including the in situ doping of Co_3_O_4_ thin films for improved catalytic performances.

## Supplementary Material



## Notes

Experimental setup images and experimental details of varying parameters; Raman spectra of varying conditions and Raman characterization details (FWHM deconvolution of peaks and intensity ratio comparison); X-ray diffractograms; lattice parameter calculations, and Williamson-Hall plots; ToF-SIMS spectra of the thin films; TEM, SAED, STEM, and EELS analyses of the thin films; and chemical composition on the near surface of the thin films evaluated from the XPS spectra (PDF)

## References

[1] Han D., Ma X., Yang X., Xiao M., Sun H., Ma L., Yu X., Ge M. (2021). Metal Organic Framework-Templated Fabrication of Exposed
Surface Defect-Enriched Co3O4 Catalysts for Efficient Toluene Oxidation. J. Colloid Interface Sci..

[2] Deng X., Tüysüz H. (2014). Cobalt-Oxide-Based
Materials as Water Oxidation Catalyst:
Recent Progress and Challenges. ACS Catal..

[3] Xu J., Gao P., Zhao T. S. (2012). Non-Precious Co 3O 4 Nano-Rod Electrocatalyst for Oxygen
Reduction Reaction in Anion-Exchange Membrane Fuel Cells. Energy Environ. Sci..

[4] Cho S. B., Sim E. S., Chung Y. C. (2018). Elucidating
the Unintentional P-Type
Nature of Spinel Co3O4: A Defect Study Using Ab-Initio Calculation. J. Eur. Ceram. Soc..

[5] Koza J. A., He Z., Miller A. S., Switzer J. A. (2012). Electrodeposition of Crystalline
Co 3O 4-A Catalyst for the Oxygen Evolution Reaction. Chem. Mater..

[6] Moysiadou A., Lee S., Hsu C. S., Chen H. M., Hu X. (2020). Mechanism of Oxygen
Evolution Catalyzed by Cobalt Oxyhydroxide: Cobalt Superoxide Species
as a Key Intermediate and Dioxygen Release as a Rate-Determining Step. J. Am. Chem. Soc..

[7] Lee W. H., Han M. H., Ko Y. J., Min B. K., Chae K. H., Oh H. S. (2022). Electrode Reconstruction
Strategy for Oxygen Evolution Reaction:
Maintaining Fe-CoOOH Phase with Intermediate-Spin State during Electrolysis. Nat. Commun..

[8] Jung S., Nandi D. K., Yeo S., Kim H., Jang Y., Bae J. S., Hong T. E., Kim S. H. (2018). Phase-Controlled
Growth of Cobalt Oxide Thin Films by Atomic Layer Deposition. Surf. Coat. Technol..

[9] Chen J., Wu X., Selloni A. (2011). Electronic
Structure and Bonding Properties of Cobalt
Oxide in the Spinel Structure. Phys. Rev. B.

[10] Natarajan K., Munirathinam E., Yang T. C. K. (2021). Operando Investigation of Structural
and Chemical Origin of Co3O4Stability in Acid under Oxygen Evolution
Reaction. ACS Appl. Mater. Interfaces.

[11] Zheng Y., Liu Y., Zhou H., Huang W., Pu Z. (2018). Complete Combustion
of Methane over Co3O4 Catalysts: Influence of PH Values. J. Alloys Compd..

[12] Xu Y., Zhang F., Sheng T., Ye T., Yi D., Yang Y., Liu S., Wang X., Yao J. (2019). Clarifying
the Controversial Catalytic Active Sites of Co3O4 for the Oxygen Evolution
Reaction. J. Mater. Chem. A.

[13] Kouotou P. M., Tian Z. Y., Mundloch U., Bahlawane N., Kohse-Höinghaus K. (2012). Controlled Synthesis
of Co3O4 Spinel
with Co­(Acac)­3 as Precursor. RSC Adv..

[14] Rautiainen A., Lindblad M., Backman L. B., Puurunen R. L. (2002). Preparation of Silica-Supported
Cobalt Catalysts through Chemisorption of Cobalt­(II) and Cobalt­(III)
Acetylacetonate. Phys. Chem. Chem. Phys..

[15] Vennela A.
B., Mangalaraj D., Muthukumarasamy N., Agilan S., Hemalatha K. V. (2019). Structural
and Optical Properties of Co3O4 Nanoparticles Prepared by Sol-Gel
Technique for Photocatalytic Application. Int.
J. Electrochem. Sci..

[16] Jirátová K., Perekrestov R., Dvořáková M., Balabánová J., Topka P., Koštejn M., Olejníček J., Čada M., Hubička Z., Kovanda F. (2019). Cobalt Oxide Catalysts
in the Form
of Thin Films Prepared by Magnetron Sputtering on Stainless-Steel
Meshes: Performance in Ethanol Oxidation. Catalysts.

[17] Hansson A. N., Linderoth S., Mogensen M., Somers M. A. J. (2007). Inter-Diffusion
between Co3O4 Coatings and the Oxide Scale on Fe-22Cr. J. Alloys Compd..

[18] Guyon C., Barkallah A., Rousseau F., Giffard K., Morvan D., Tatoulian M. (2011). Deposition
of Cobalt Oxide Thin Films by Plasma-Enhanced
Chemical Vapour Deposition (PECVD) for Catalytic Applications. Surf. Coat. Technol..

[19] Burriel M., Garcia G., Santiso J., Hansson A. N., Linderoth S., Figueras A. (2005). Co3O4 Protective Coatings
Prepared by Pulsed Injection
Metal Organic Chemical Vapour Deposition. Thin
Solid Films.

[20] Maurau R., Boscher N. D., Olivier S., Bulou S., Belmonte T., Dutroncy J. Ô., Sindzingre T., Choquet P. (2013). Atmospheric Pressure,
Low Temperature Deposition of Photocatalytic TiOx Thin Films with
a Blown Arc Discharge. Surf. Coat. Technol..

[21] Chemin J. B., Bulou S., Baba K., Fontaine C., Sindzingre T., Boscher N. D., Choquet P. (2018). Transparent
Anti-Fogging and Self-Cleaning
TiO2/SiO2 Thin Films on Polymer Substrates Using Atmospheric Plasma. Sci. Rep..

[22] Mariotti D., Belmonte T., Benedikt J., Velusamy T., Jain G., Švrček V. (2016). Low-Temperature Atmospheric Pressure Plasma Processes
for “Green” Third Generation Photovoltaics. Plasma Process. Polym..

[23] Huerta-Flores A. M., Usiobo O. J., Audinot J. N., Heyberger R., Choquet P., Boscher N. D. (2022). Low Temperature
Open-Air Plasma Deposition
of SrTiO3Films for Solar Energy Harvesting: Impact of Precursors on
the Properties and Performances. ACS Appl. Mater.
Interfaces.

[24] Moravej M., Hicks R. F. (2005). Atmospheric Plasma Deposition of Coatings Using a Capacitive
Discharge Source. Chem. Vap. Deposition.

[25] Merche D., Vandencasteele N., Reniers F. (2012). Atmospheric Plasmas for Thin Film
Deposition: A Critical Review. Thin Solid Films.

[26] Uricchio A., Fanelli F. (2021). Low-Temperature Atmospheric
Pressure Plasma Processes
for the Deposition of Nanocomposite Coatings. Processes.

[27] Ondo D. A., Loyer F., Boscher N. D. (2021). Influence of Double Bonds and Cyclic
Structure on the AP-PECVD of Low-k Organosilicon Insulating Layers. Plasma Processes Polym..

[28] Knapp C. E., Metcalf E. A., Mrig S., Sanchez-Perez C., Douglas S. P., Choquet P., Boscher N. D. (2018). Precursors for Atmospheric
Plasma-Enhanced Sintering: Low-Temperature Inkjet Printing of Conductive
Copper. ChemistryOpen.

[29] Massines F., Sarra-Bournet C., Fanelli F., Naudé N., Gherardi N. (2012). Atmospheric Pressure
Low Temperature Direct Plasma
Technology: Status and Challenges for Thin Film Deposition. Plasma Processes Polym..

[30] Belmonte T., Henrion G., Gries T. (2011). Nonequilibrium Atmospheric Plasma
Deposition. J. Therm. Spray Technol..

[31] Snyders R., Hegemann D., Thiry D., Zabeida O., Klemberg-Sapieha J., Martinu L. (2023). Foundations of Plasma
Enhanced Chemical Vapor Deposition
of Functional Coatings. Plasma Sources Sci.
Technol..

[32] Takeda K., Ishikawa K., Tanaka H., Sekine M., Hori M. (2017). Spatial Distributions
of O, N, NO, OH and Vacuum Ultraviolet Light along Gas Flow Direction
in an AC-Excited Atmospheric Pressure Ar Plasma Jet Generated in Open
Air. J. Phys. D Appl. Phys..

[33] Johnson K. W., Guruvenket S., Sailer R. A., Ahrenkiel S. P., Schulz D. L. (2013). Atmospheric Pressure
Plasma Enhanced Chemical Vapor
Deposition of Zinc Oxide and Aluminum Zinc Oxide. Thin Solid Films.

[34] Lalancette R. A., Syzdek D., Grebowicz J., Arslan E., Bernal I. (2019). The Thermal
Decomposition and Analyses of Metal Tris-Acetylacetonates: Free Radical
Formation from Al, Cr, Mn, Fe and Co Complexes. J. Therm. Anal. Calorim..

[35] Schubert J. S., Popovic J., Haselmann G. M., Nandan S. P., Wang J., Giesriegl A., Cherevan A. S., Eder D. (2019). Immobilization of Co,
Mn, Ni and Fe Oxide Co-Catalysts on TiO2 for Photocatalytic Water
Splitting Reactions. J. Mater. Chem. A.

[36] Berry A. D., Gaskill D. K., Holm R. T., Cukauskas E. J., Kaplan R., Henry R. L. (1988). Formation of High
Tc Superconducting
Films by Organometallic Chemical Vapor Deposition. Appl. Phys. Lett..

[37] Cardenas-Flechas L. J., Barba-Ortega J. J., Joya M. R. (2022). Analysis and Evaluation of Structural
Properties of Co3O4Microparticles Obtained at Low Temperature. Ceramica.

[38] Hadjiev V. G., Iliev M. N., Vergilov I. V. (1988). The Raman
Spectra of Co_3_O_4_. J. Phys.
C: Solid State Phys..

[39] Tyczkowski J., Kapica R., Łojewska J. (2007). Thin Cobalt
Oxide Films for Catalysis
Deposited by Plasma-Enhanced Metal-Organic Chemical Vapor Deposition. Thin Solid Films.

[40] Laguna-Bercero M. A., Sanjuán M. L., Merino R. I. (2007). Raman Spectroscopic Study of Cation
Disorder in Poly- and Single Crystals of the Nickel Aluminate Spinel. J. Phys.: Condens. Matter.

[41] Wu M., Chen S., Soomro A., Ma S., Zhu M., Hua X., Xiang W. (2019). Investigation of Synergistic
Effects and High Performance
of La-Co Composite Oxides for Toluene Catalytic Oxidation at Low Temperature. Environ. Sci. Pollut. Res..

[42] Gao L., Yalon E., Chew A. R., Deshmukh S., Salleo A., Pop E., Demkov A. A. (2017). Effect
of Oxygen Vacancies and Strain on the Phonon
Spectrum of HfO2 Thin Films. J. Appl. Phys..

[43] Chen Z., Kronawitter C. X., Koel B. E. (2015). Facet-Dependent Activity and Stability
of Co3O4 Nanocrystals towards the Oxygen Evolution Reaction. Phys. Chem. Chem. Phys..

[44] Xiao Z., Huang Y. C., Dong C. L., Xie C., Liu Z., Du S., Chen W., Yan D., Tao L., Shu Z., Zhang G., Duan H., Wang Y., Zou Y., Chen R., Wang S. (2020). Operando Identification of the Dynamic
Behavior of Oxygen Vacancy-Rich Co3O4for Oxygen Evolution Reaction. J. Am. Chem. Soc..

[45] Chen G. Y., Zhu S. L., Han X. Q., Wang D. C., Zhang J. C., Huai X. D., Li X., Zhang F. Y., Xiang Z., Zhang W. Z. (2023). Engineering Cationic
Vacancies in Octahedral Sites
of Co3O4for High-Efficiency Oxygen Evolution. Energy Fuels.

[46] Wu J., Xue Y., Yan X., Yan W., Cheng Q., Xie Y. (2012). Co3O4 Nanocrystals
on Single-Walled Carbon Nanotubes as a Highly Efficient Oxygen-Evolving
Catalyst. Nano Res..

[47] Saddeler S., Hagemann U., Schulz S. (2020). Effect of
the Size and Shape on the
Electrocatalytic Activity of Co3O4Nanoparticles in the Oxygen Evolution
Reaction. Inorg. Chem..

[48] Biesinger M. C., Payne B. P., Grosvenor A. P., Lau L. W. M., Gerson A. R., Smart R. S. C. (2011). Resolving Surface
Chemical States in XPS Analysis of
First Row Transition Metals, Oxides and Hydroxides: Cr, Mn, Fe, Co
and Ni. Appl. Surf. Sci..

[49] Burke M. S., Kast M. G., Trotochaud L., Smith A. M., Boettcher S. W. (2015). Cobalt-Iron
(Oxy)­Hydroxide Oxygen Evolution Electrocatalysts: The Role of Structure
and Composition on Activity, Stability, and Mechanism. J. Am. Chem. Soc..

[50] Bergmann A., Martinez-Moreno E., Teschner D., Chernev P., Gliech M., De Araújo J. F., Reier T., Dau H., Strasser P. (2015). Reversible
Amorphization and the Catalytically Active State of Crystalline Co3O4
during Oxygen Evolution. Nat. Commun..

[51] Muñoz-Ferreiro C., López-Pernía C., Gallardo-López Á., Poyato R. (2021). Unravelling the Optimization of Few-Layer Graphene
Crystallinity and Electrical Conductivity in Ceramic Composites by
Raman Spectroscopy. J. Eur. Ceram. Soc..

[52] Pearse, R. W. B. ; Gaydon, A. G. The Identification of Molecular Spectra, 4th ed.; Chapman; Hall , Eds.; John Wiley & Sons, Inc: London, 1976; Vol. 297.

[53] Luque, J. ; Crosley, D. R. LIFBASE: Database and Spectral Simulation Program (Version 1.5). 1999.

[54] Gessel, A. F. H. ; Bruggeman; Van Gessel, A. ; Hrycak, B. ; Jasí Nski, M. ; Mizeraczyk, J. ; Van Der Mullen, J. ; Bruggeman, P. J. Temperature and NO Density Measurements by LIF and OES on an Atmospheric Pressure Plasma Jet Citation for Published Version (APA): Temperature and NO Density Measurements by LIF and OES on an Atmospheric Pressure Plasma Jet J. Phys. D: Appl. Phys., 46 9 445102 10.1088/0022.

[55] Acharya K., Bulou S., Gaulain T., Choquet P. (2021). AP-PACVD Plasma Printer:
Investigating the Influence of Gas Flow Rates to Printing Resolution
in Parallel with CFD Simulation. J. Phys. D:
Appl. Phys..

[56] Griem, H. R. Principles of Plasma Spectroscopy 2005.

[57] Bardos L. (1988). Afterglow
and Decaying Plasma CVD Systems. Vacuum.

[58] Brisset J. L., Moussa D., Doubla A., Hnatiuc E., Hnatiuc B., Kamgang Youbi G., Herry J. M., Naïtali M., Bellon-Fontaine M. N. (2008). Chemical Reactivity of Discharges and Temporal Post-Discharges
in Plasma Treatment of Aqueous Media: Examples of Gliding Discharge
Treated Solutions. Ind. Eng. Chem. Res..

[59] Bae J. H., Huh S. C., Park J. Y., Park S., Eom S., Ryu S., Lee H., Park S. (2024). Lifetime of Nitric Oxide Produced
by Surface Dielectric Barrier Discharge in Controlled Atmospheres:
Role of O2 Content. Chem. Eng. J. Adv..

[60] Guerra V., Sá P. A., Loureiro J. (2001). Role Played by the N 2 (A 3 ∑
u + ) Metastable in Stationary N 2 and N 2-O 2 Discharges You May
Also like Role Played by the N 2 (A 3 ∑ + u) Metastable in
Stationary N 2 and N 2-O 2 Discharges. J. Phys.
D: Appl. Phys..

[61] Mozetič, M. ; Vesel, A. ; Primc, G. ; Zaplotnik, R. Introduction to Plasma and Plasma Diagnostics. In Non-Thermal Plasma Technology for Polymeric Materials: Applications in Composites, Nanostructured Materials, and Biomedical Fields; Elsevier, 2018; pp 23–65.

[62] Fukuchi T. (2010). Detection
of Metastable Excited Molecules N2­(A 3∑u+) in an Atmospheric
Pressure Nitrogen Discharge by Raman Scattering. Electron. Commun. Jpn..

[63] Hilt F., Hovish M. Q., Rolston N., Brüning K., Tassone C. J., Dauskardt R. H. (2018). Rapid Route
to Efficient, Scalable,
and Robust Perovskite Photovoltaics in Air. Energy Environ. Sci..

[64] Snyders R., Hegemann D., Thiry D., Zabeida O., Klemberg-Sapieha J., Martinu L. (2023). Foundations of Plasma
Enhanced Chemical Vapor Deposition
of Functional Coatings. Plasma Sources Sci.
Technol..

[65] Abuyazid N. H., Üner N. B., Peyres S. M., Mohan
Sankaran R. (2023). Charge Decay
in the Spatial Afterglow of Plasmas and Its Impact on Diffusion Regimes. Nat. Commun..

[66] Huber, K. P. ; Herzberg, G. Molecular Spectra and Molecular Structure; Springer: US, 1979.

[67] Guruvenket S., Andrie S., Simon M., Johnson K. W., Sailer R. A. (2012). Atmospheric-Pressure
Plasma-Enhanced Chemical Vapor Deposition of a-SiCN:H Films: Role
of Precursors on the Film Growth and Properties. ACS Appl. Mater. Interfaces.

[68] Reuter R., Ellerweg D., Von Keudell A., Benedikt J. (2011). Surface Reactions as
Carbon Removal Mechanism in Deposition of Silicon Dioxide Films at
Atmospheric Pressure. Appl. Phys. Lett..

[69] Ghobeira R., Esbah Tabaei P. S., Nikiforov A., Morent R., De Geyter N. (2023). Unraveling
Exclusive In-Plasma Initiated Oxidation Processes Occurring at Polymeric
Surfaces upon O2 Admixtures to Medium Pressure Ar and N2 DBD Treatments. Polymers.

[70] Luo, Y.-R. ; Kerr, J. A. Bond Dissociation Energies. In CRC Handbook of Chemistry and Physics; CRC Press, 2012; Vol. 8989, pp 65–98.

