# Supplementary material for: Cobalt Oxide (Co3O4) Thin Films Synthesized by Atmospheric Pressure PECVD: Deposition Mechanisms and Catalytic Potential
Source: ACS Appl Energy Mater. 2025 May 29;8(11):7038–51. doi: 10.1021/acsaem.5c00280 (PMC12152839; doi:10.1021/acsaem.5c00280)
Supplement: Supplementary file 1 [file ae5c00280_si_001.pdf]

Supporting Information

Cobalt oxide (Co_3O_4) thin films synthesized by atmospheric pressure PECVD: Deposition mechanisms and catalytic potential

João Mallmann*[,1,2], Jean-Baptiste Chemin[1], Drialys Cardenas Morcoso[1], Adrian-Marie Philippe[3], Simon Bulou[1], Nihed Chaabane[4], Fabien Rouillard[2], Patrick Choquet[1] and Nicolas D. Boscher*[,1]

[1] *Luxembourg Institute of Science and Technology, Advanced Plasma and Vapor Deposition Processes Engineering, L-4362, Esch-sur-Alzette, Luxembourg*

[2] *CEA, Service de Recherche en Corrosion et Comportement des Matériaux, Université Paris Saclay, 91191 Gif-sur-Yvette, France*

[3] *Luxembourg Institute of Science and Technology, Advanced Characterization of Surface, Interface and Structure, L-4422, Belvaux, Luxembourg*

[4] *CEA, Institut National des Sciences et Techniques Nucléaires, Université Paris Saclay, 91191 Gif-sur-Yvette, France*

E-mail: joamallmann@gmail.com

E-mail: nicolas.boscher@list.lu

Table of Contents

Figure S1. X-ray Diffractograms of the samples produced with no additional heating, at 250°C and 300°C with 40% O_2 concentration in the carrier gas (a). Variation of $\sin^2\theta$ versus ($h^2+k^2+l^2$) for the Co_3O_4 thin films prepared without additional heating system (light blue), at 250°C (blue) and at 300°C (dark blue). The slope of the linear fit (A) was applied in equation ($A = \lambda/4a^2$) to determine the lattice parameter 8.08 Å, 8.078 Å, and 8.074 Å, respectively (b). Williamson-Hall plot of the sample produced at 250°C with 40% O_2 concentration in the carrier gas (c)……………………………………………..**S3**

Figure S2. Cyclic voltammogram recorded at 100 mV s^{-1}, in 1 M KOH for the bare FTO reference sample. No electrocatalytic potential………………………………………………………………**S4**

Table S1. Deposition parameters used during the AP-PECVD process for two different conditions…**S4**

Figure S3. SEM cross-section images of the samples produced in open-air at 250°C and 40% O_2 (a), 0% O_2 (b), 60% O_2 (c), and samples produced in open-air using 40% O_2 without a heating system (d) and at 300°C (e)………………………………………………………………………………**S5**

Figure S4. TEM cross section of the Co_3O_4 thin film (a). Selected area electron diffraction (SAED) image (b) corresponding to the region highlighted in the orange dotted circle. The SAED analysis confirms the formation of crystalline Co_3O_4. Peak positions are the same as found in XRD. There is a slight deviation from the theoretical value for Co_3O_4. High Angle Annular Dark Field (HAADF) STEM image (c) highlights the variation in chemical composition. Darker regions correspond to voids or lighter elements, i.e., carbon……………………………………………………………………..**S5**

Figure S5. High Angle Annular Dark Field (HAADF) STEM image (a) shows the presence of dark regions in the thin film. The region in the dotted yellow square was analysed by Electron Energy Loss Spectroscopy (EELS). The Co, O and C mapping of this region is shown in (b), while the spectra of regions 1 and 2 are shown in (c)……………………………………………………………………**S6**

Figure S6. Raman spectra of the Co_3O_4 thin films at different O_2 concentrations (0%, 20%, 40% and 60%) in the carrier gas compared to the Co_3O_4 reference (magenta). Lack of variation on peak position irrespective of the O_2 concentration. In all cases, there is a good agreement with the Co_3O_4 reference sample………………………………………………………………………………………**S6**

Figure S7. Carbon and nitrogen concentrations determined by XPS analysis after 900 s of Ar_{1000}^+ cluster sputtering for the thin film prepared under open-air conditions and for different concentration of O_2 in the carrier gas (250°C as substrate heating temperature) (a). Carbon concentration in function of the substrate heating temperature (40% O_2 in the carrier gas) (b) determined after 900 s of Ar_{1000}^+ cluster sputtering……………………………………………………………………………………...**S7**

Figure S8. Raman spectra of the Co_3O_4 thin films at different heating temperatures (no heating, 200°C, 250°C and 300°C) compared to the $Co(acac)_3$ (black) precursor and the Co_3O_4 reference (magenta). Lack of variation on peak position irrespective of the temperature. In all cases, there is a good agreement with the Co_3O_4 reference sample, and no evidence of the precursor…………………………………**S7**

Table S2. Peak positions, FWHM for the peaks F_2g (1) and A_1g, which refers to Co^{2+} and Co^{3+} respectively, and the peak ratio based on the intensity of these two peaks. Data was acquired from the Raman spectrum for all conditions and FWHM was calculated using a Lorentzian deconvolution from Origin software. The data was compared to a reference from literature (Hadjiev et al.) and to a Co_3O_4 standard produced in the laboratory……………………………………………………………………..**S8**

Figure S9. ToF-SIMS analysis of the Co_3O_4 thin films produced without additional heating (right) and at 300 °C (left). In both cases, it is evident the steep decrease in residual impurities (OH^- and CO_3^-) after the initial sputtering. Additionally, Co^+ and O^- remain stable along the thin film……………………**S9**

Figure S10. Experimental setup for the test under inert atmosphere. The plasma torch was placed in an acrylic box. The O_2 detector was placed inside the box to measure the O_2 concentration (red arrow). Prior to performing the deposition, N_2 was purged to ensure low O_2 concentration………………...**S10**

Figure S11. Plasma color variation on the open-air (left) and inert atmosphere (right). The color change highlights the lack of O_2 in the inert atmosphere……………………………………………………**S10**

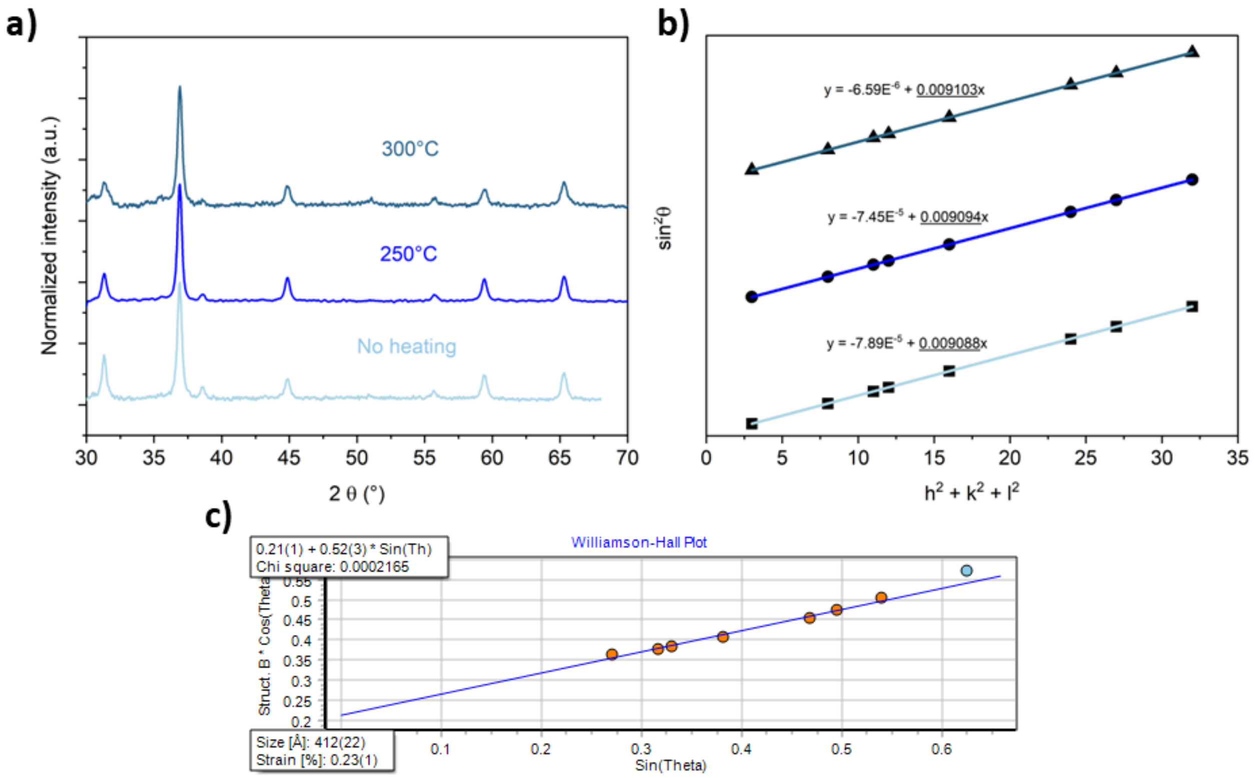

Figure S1. X-ray Diffractograms of the samples produced with no additional heating, at 250°C and 300°C with 40% O_2 concentration in the carrier gas (a). Variation of $\sin^2\theta$ versus $(h^2+k^2+l^2)$ for the Co_3O_4 thin films prepared without additional heating system (light blue), at 250°C (blue) and at 300°C (dark blue). The slope of the linear fit (A) was applied in equation ($A = \lambda/4a^2$) to determine the lattice parameter 8.08 Å, 8.078 Å, and 8.074 Å, respectively (b). Williamson-Hall plot of the sample produced at 250°C with 40% O_2 concentration in the carrier gas (c).

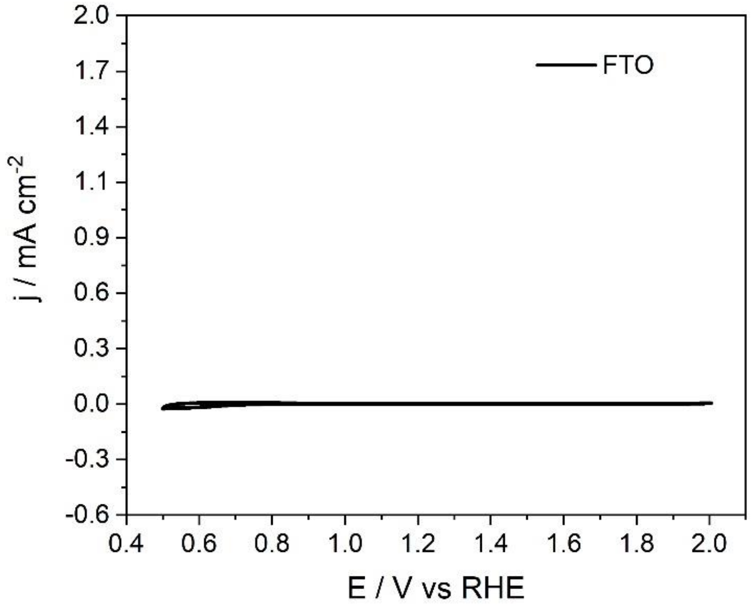

Figure S2. Cyclic voltammogram recorded at 100 mV s^{-1}, in 1 M KOH for the bare FTO reference sample. No electrocatalytic potential.

Table S1. Deposition parameters used during the AP-PECVD process for two different conditions.

Parameters	Inert atmosphere	Open-air
Plasma gas	Nitrogen (50 slm)	Nitrogen (50 slm)
Plasma power	1000 W	1000 W
Atomising gas	N_2 (2 slm)	N_2 (2 slm)
Carrier gas	N_2 (2 slm)	N_2 (0.8-2 slm) + O_2 (0-1.2 slm)
Precursor concentration	10 mM	10 mM
Precursor injection rate	400 µL/min	400 µL/min
Displacement speed	Static	0.05 mm/s
Substrate heating temperature	No heating	No heating – 300 °C
Injection distance (from plasma)	15 mm	15 mm
Substrate position (from) injection point	1 mm	1 mm

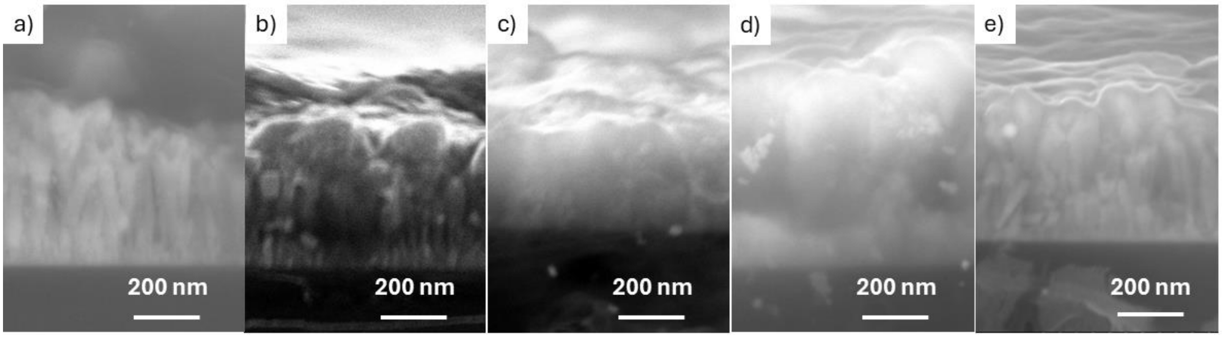

Figure S3. SEM cross-section images of the samples produced in open-air at 250°C and 40% O_2 (a), 0% O_2 (b), 60% O_2 (c), and samples produced in open-air using 40% O_2 without a heating system (d) and at 300°C (e).

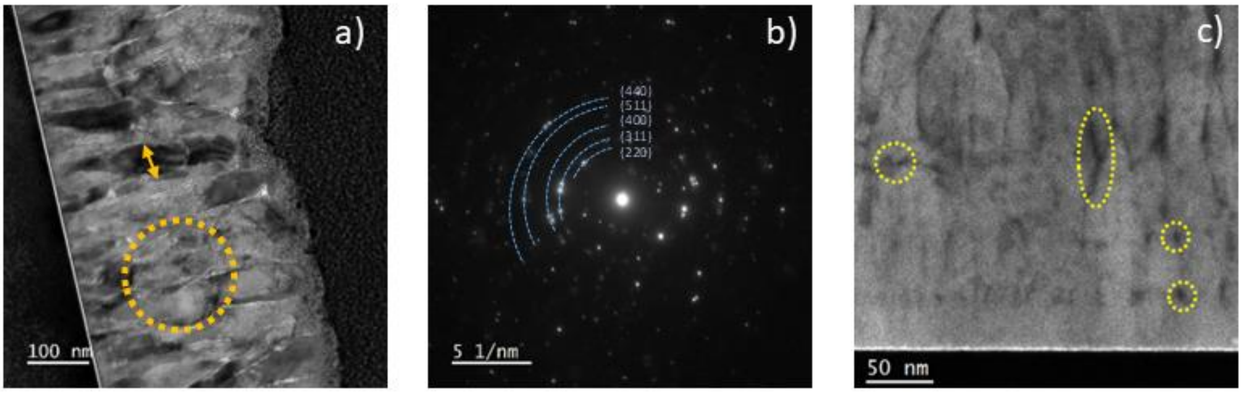

Figure S4. TEM cross section of the Co_3O_4 thin film (a). Selected area electron diffraction (SAED) image (b) corresponding to the region highlighted in the orange dotted circle. The SAED analysis confirms the formation of crystalline Co_3O_4. Peak positions are the same as found in XRD. There is a slight deviation from the theoretical value for Co_3O_4. High Angle Annular Dark Field (HAADF) STEM image (c) highlights the variation in chemical composition. Darker regions correspond to voids or lighter elements, i.e., carbon.

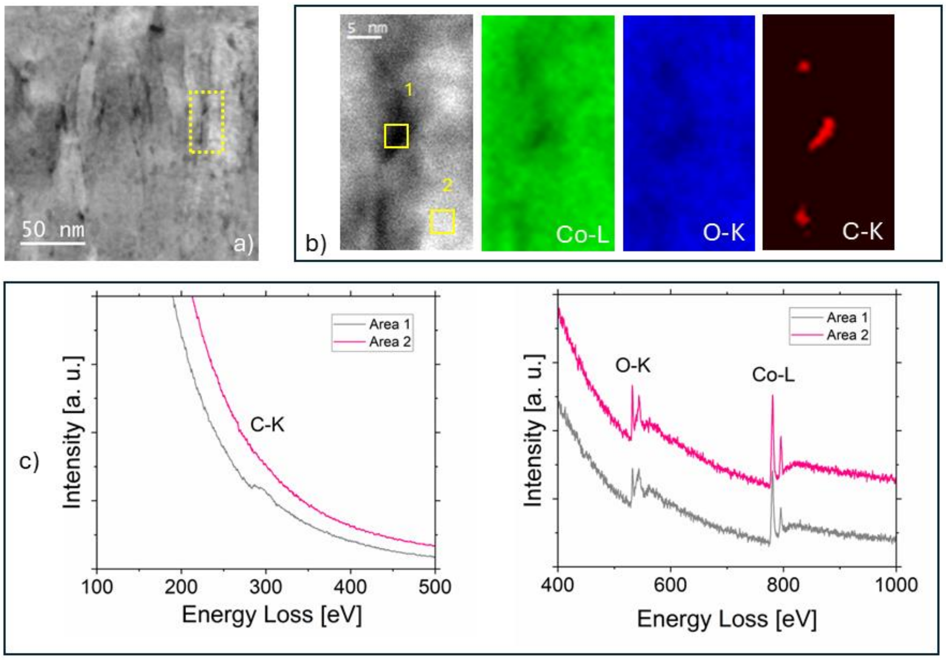

Figure S5. High Angle Annular Dark Field (HAADF) STEM image (a) shows the presence of dark regions in the thin film. The region in the dotted yellow square was analysed by Electron Energy Loss Spectroscopy (EELS). The Co, O and C mapping of this region is shown in (b), while the spectra of regions 1 and 2 are shown in (c).

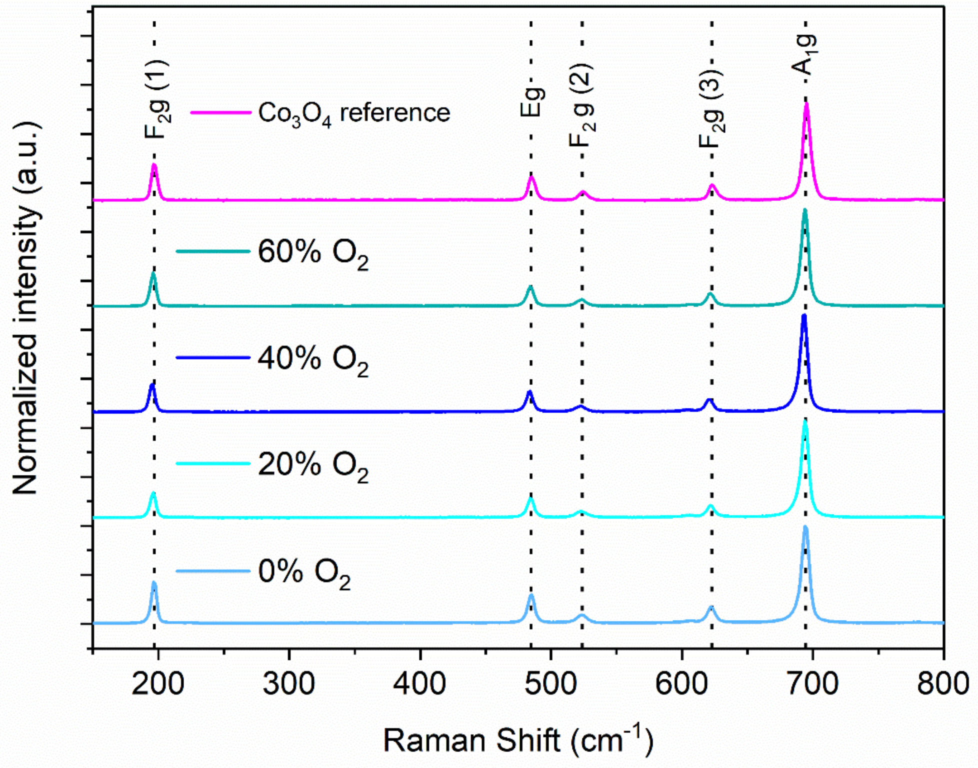

Figure S6. Raman spectra of the Co_3O_4 thin films at different O_2 concentrations (0%, 20%, 40% and 60%) in the carrier gas compared to the Co_3O_4 reference (magenta). Lack of variation on peak position irrespective of the O_2 concentration. In all cases, there is a good agreement with the Co_3O_4 reference sample.

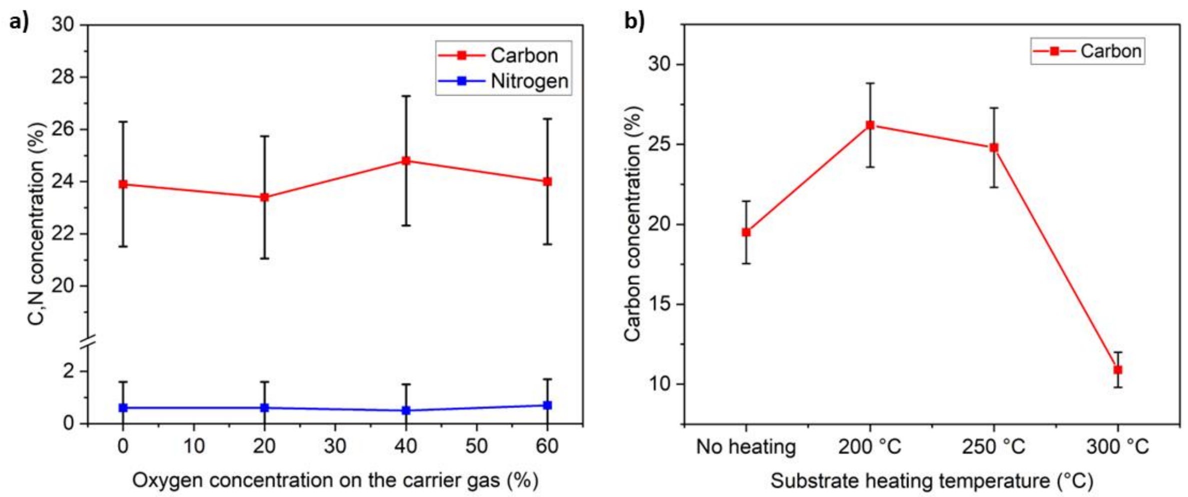

Figure S7. Carbon and nitrogen concentrations determined by XPS analysis after 900 s of Ar_{1000}^{+} cluster sputtering for the thin film prepared under open-air conditions and for different concentration of O_2 in the carrier gas (250°C as substrate heating temperature) (a). Carbon concentration in function of the substrate heating temperature (40% O_2 in the carrier gas) (b) determined after 900 s of Ar_{1000}^{+} cluster sputtering.

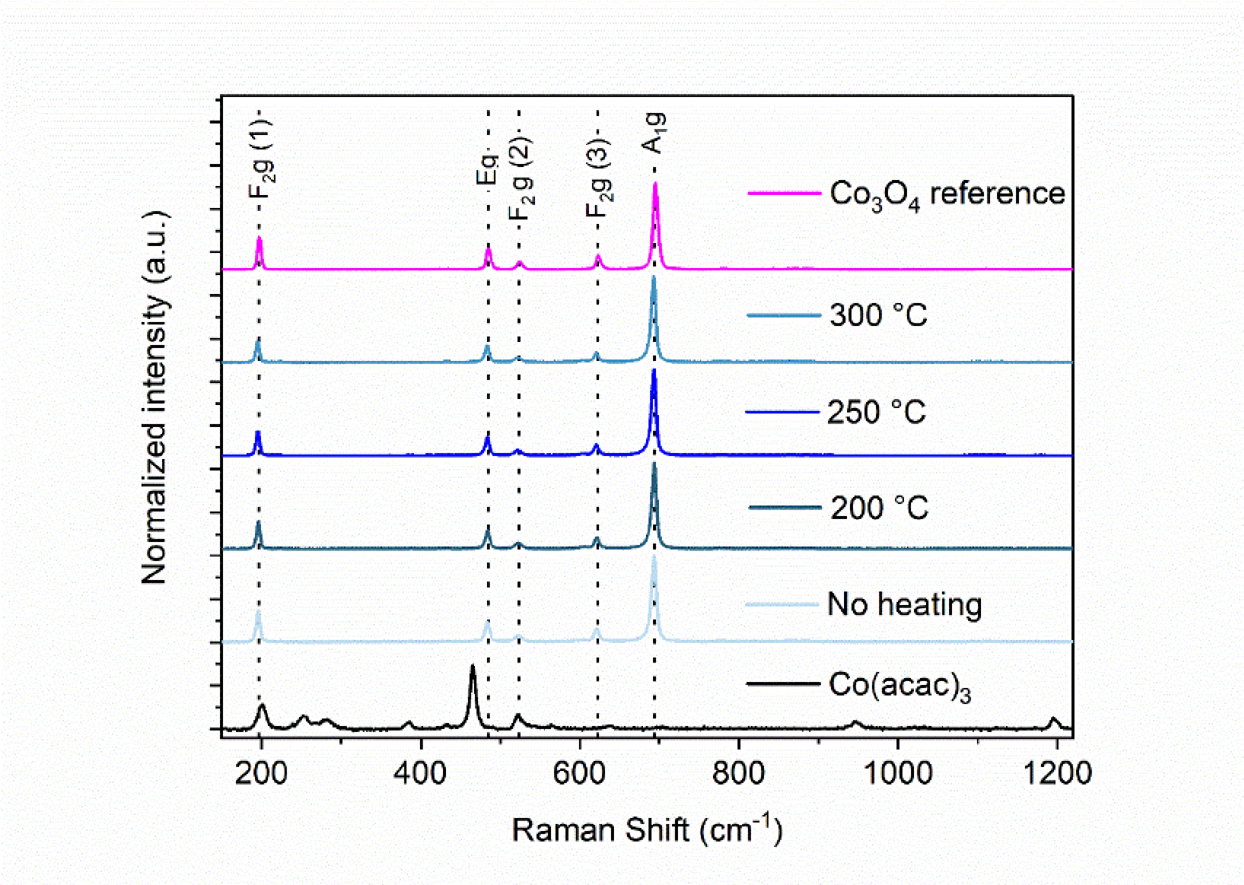

Figure S8. Raman spectra of the Co_3O_4 thin films at different heating temperatures (no heating, 200°C, 250°C and 300°C) compared to the $Co(acac)_3$ (black) precursor and the Co_3O_4 reference (magenta). Lack of variation on peak position irrespective of the temperature. In all cases, there is a good agreement with the Co_3O_4 reference sample, and no evidence of the precursor.

Table S2. Peak positions, FWHM for the peaks F_2g (1) and A_1g, which refers to Co^{2+} and Co^{3+} respectively, and the peak ratio based on the intensity of these two peaks. Data was acquired from the Raman spectrum for all conditions and FWHM was calculated using a Lorentzian deconvolution from Origin software. The data was compared to a reference from literature (Hadjiev et al.) and to a Co_3O_4 standard produced in the laboratory.

Sample	**F_2g (1) (cm^{-1})**	**FWHM (cm^{-1})**	**Peak ratio ($F2g/A_1g$)**	**Eg (cm^{-1})**	**F_2g (2) (cm^{-1})**	**F_2g (3) (cm^{-1})**	**A_1g (cm^{-1})**	**FWHM (cm^{-1})**
Hadjiev et al.	194.4	4.9		482.4	521.6	618.4	691	6.2
No Heating – 40% O_2	195.5	4.5	0.36	483.8	522.6	621.2	693.3	6.7
200°C – 40% O_2	195.7	4.4	0.32	484	522.7	621.4	693.5	6.3
250°C – 40% O_2	195.2	4.3	0.29	483.5	522.2	620.8	693	6.15
300°C – 40% O_2	194.9	4.4	0.26	483.3	522.1	620.6	692.8	6.18
**Co_3O_4 REFERENCE (current work)**	**197**	**3.6**	**0.37**	**484.7**	**523.7**	**622.7**	**694.6**	**4.8**
250°C – 0% O_2	196.6	4.07	0.43	484.7	523.4	622.2	694.2	6.5
250°C - 20% O_2	195.8	4.8	0.26	484.3	522.98	621.8	693.8	6.36
250°C – 40% O_2	195.2	4.3	0.29	483.5	522.2	620.8	693	6.15
250°C – 60% O_2	196	4.4	0.35	484.1	523	621.7	693.7	6.28

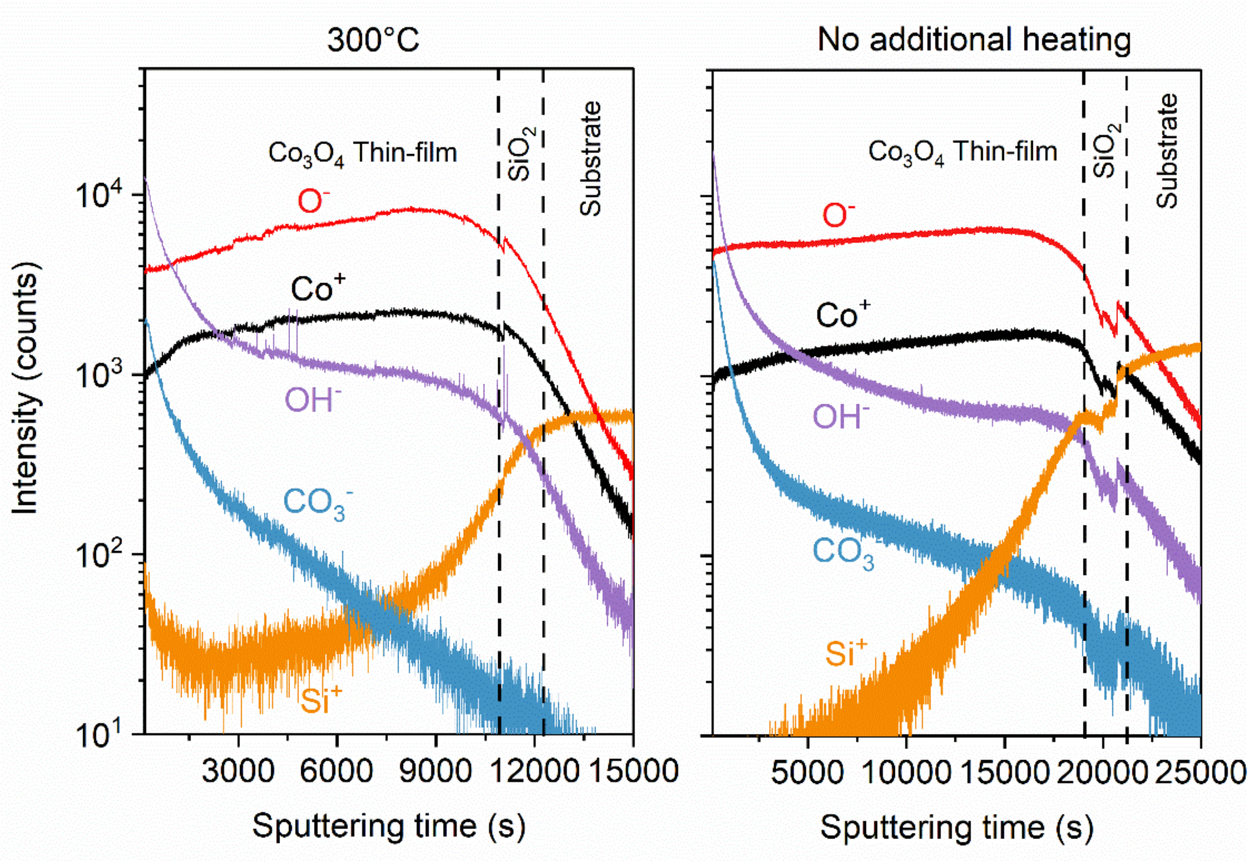

Figure S9. ToF-SIMS analysis of the Co_3O_4 thin films produced without additional heating (right) and at 300 °C (left). In both cases, it is evident the steep decrease in residual impurities (OH^- and CO_3^-) after the initial sputtering. Additionally, Co^+ and O^- remain stable along the thin film.

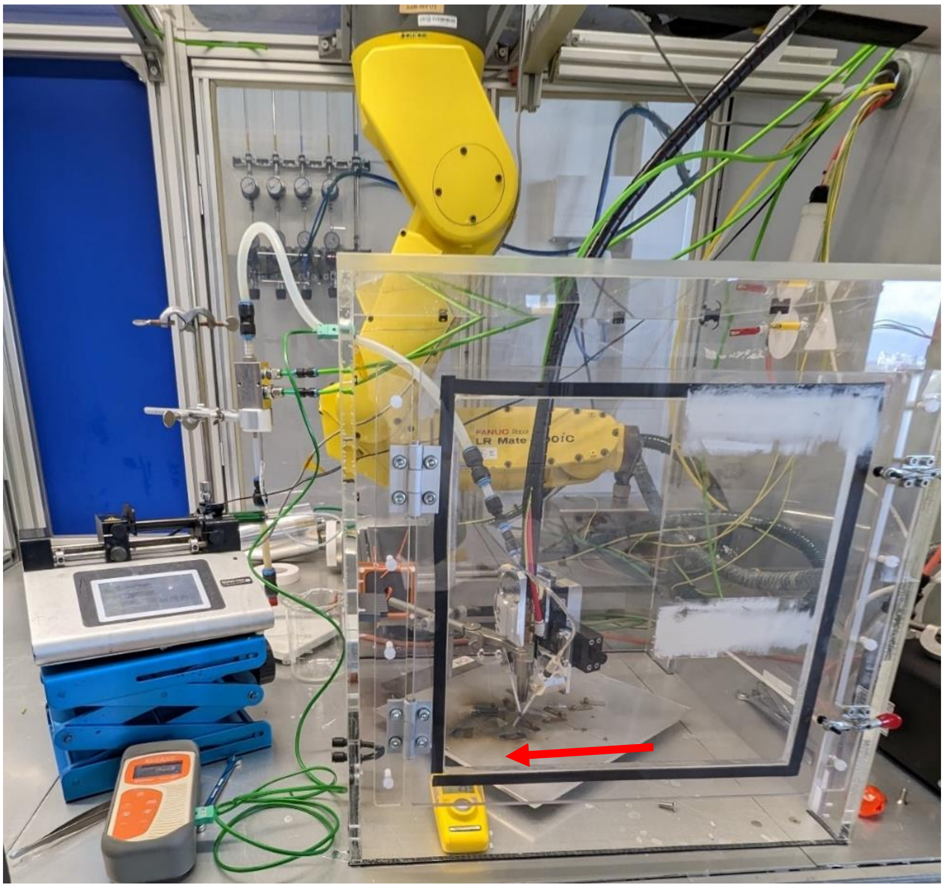

Figure S10. Experimental setup for the test under inert atmosphere. The plasma torch was placed in an acrylic box. The O_2 detector was placed inside the box to measure the O_2 concentration (red arrow). Prior to performing the deposition, N_2 was purged to ensure low O_2 concentration.

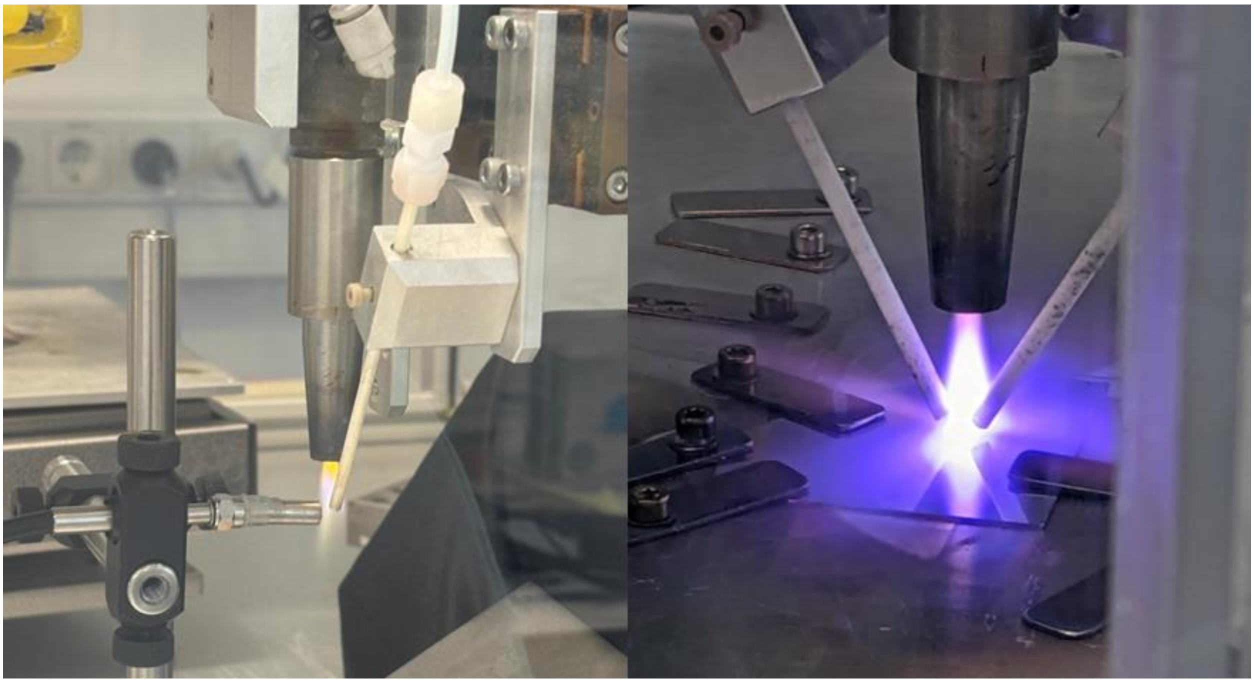

Figure S11. Plasma color variation on the open-air (left) and inert atmosphere (right). The color change highlights the lack of O_2 in the inert atmosphere.
